# Exposure to urban nanoparticles at low PM$_1$ concentrations as a source of oxidative stress and inflammation

**DOI:** 10.1038/s41598-023-45230-z

**Published:** 2023-10-30

**Authors:** Francesca Costabile, Maurizio Gualtieri, Matteo Rinaldi, Silvia Canepari, Roberta Vecchi, Lorenzo Massimi, Gianluca Di Iulio, Marco Paglione, Luca Di Liberto, Emanuela Corsini, Maria Cristina Facchini, Stefano Decesari

**Affiliations:** 1 Institute of Atmospheric Sciences and Climate - Italian National Research Council (ISAC - CNR), Via Fosso del Cavaliere, 00133 Rome, Italy; 2 National Biodiversity Future Center, NBFC, 90133 Palermo, Italy; 3 Department of Earth and Environmental Sciences, University of Milano-Bicocca, 26126 Milan, Italy; 4 Institute of Atmospheric Sciences and Climate - Italian National Research Council (ISAC - CNR), Via Gobetti, 40129 Bologna, Italy; 5 Department of Environmental Biology, University of Rome Sapienza, 00185 Rome, Italy; 6 Department of Physics, Università degli Studi di Milano,and INFN-Milan, 20133 Milan, Italy; 7 Department of Pharmacological and Biomolecular Sciences, Università degli Studi di Milano, 20133 Milan, Italy

**Keywords:** Environmental sciences, Environmental chemistry, Environmental impact

## Abstract

Exposures to fine particulate matter (PM$_1$) have been associated with health impacts, but the understanding of the PM$_1$ concentration-response (PM$_1$-CR) relationships, especially at low PM$_1$, remains incomplete. Here, we present novel data using a methodology to mimic lung exposure to ambient air (2$<PM_1<$ 60 $\upmu$g m$^{-3}$), with minimized sampling artifacts for nanoparticles. A reference model (Air Liquid Interface cultures of human bronchial epithelial cells, BEAS-2B) was used for aerosol exposure. Non-linearities observed in PM$_1$-CR curves are interpreted as a result of the interplay between the aerosol total oxidative potential (OP$_t$) and its distribution across particle size (d$_p$). A d$_p$-dependent condensation sink (CS) is assessed together with the distribution with d$_p$ of reactive species . Urban ambient aerosol high in OP$_t$, as indicated by the DTT assay, with (possibly copper-containing) nanoparticles, shows higher pro-inflammatory and oxidative responses, this occurring at lower PM$_1$ concentrations (< 5 $\upmu$g m$^{-3}$). Among the implications of this work, there are recommendations for global efforts to go toward the refinement of actual air quality standards with metrics considering the distribution of OP$_t$ with d$_p$ also at relatively low PM$_1$.

## Introduction

In the past decade, much research has focused on the understanding of how human and planetary health is threatened by the fine particulate matter (PM$_{2.5}$, particles with aerodynamic diameter smaller than 2.5 $\upmu$m) in the atmosphere^1–6^. A large body of epidemiological evidence supports a causal relationship between long-term exposure to outdoor PM$_{2.5}$ and premature mortality^3,7–9^. However, it remains challenging to create a comprehensive model explaining the varying toxicological impact of each $\upmu$g of PM$_{2.5}$ in the air^1,5,6,10,11^. Recently, the WHO guidelines for outdoor PM$_{2.5}$ addressed, in particular, the low range of PM$_{2.5}$ concentrations (< 5 $\upmu$g m$^{-3}$, i.e. below actual air quality standards) . These guidelines reflect the near-background global PM$_{2.5}$ concentrations. This is the range where the understanding of the concentration-response (CR) relationship for PM$_{2.5}$ and mortality remains largely incomplete^7,8,12,13^. Evidence is to be sought by identifying a causal association combined with a set of plausible toxicological mechanisms and intermediate endpoints by which PM$_{2.5}$ could cause adverse health effects.

There is a growing scientific consensus that the capacity of inhaled PM$_{2.5}$ to induce oxidative stress and inflammation in exposed populations may be a primary pathway leading to the development of cardiovascular disease outcomes^11,14–16^. Among the mechanisms possibly associated with adverse health outcomes, there is the initiation of inflammatory response within the conducting airways via pro-oxidative and/or pro-inflammatory mediators^17^; translocation of nanometer-size particles (or ultrafine particles, UFPs, diameter < 100 nm) and/or particle constituents such as organic compounds and metals across the alveolar barrier^18,19^, as well as indirect responses triggered by the activation of airways sensory nerves and, in cascade misbalancing the autonomic nervous system imbalance^20^. For each of these pathways, a cellular oxidative imbalance between the generation and elimination of reactive oxygen species (ROS) can potentially occur^14^. This is clearly depending upon the nature of the atmospheric aerosols (the interplay between their particle size and components versus gases and vapors), in a complex fashion which is still to be elucidated^21,22^. Synergistic effects can occur among different physical and chemical properties. The so-called Trojan-horse mechanism has been proposed to enhance the intracellular release of toxics if bound to nanometer-sized particles, and hence the damaging action of partially soluble materials due to the partial solubilization of these particles within cells^16,22,23^. This mechanism is likely to act also for atmospheric aerosols. Indeed, the solid core of ultrafine particles may act as a carrier for inorganic and organic species as reported for classical carbon nanotubes^24^ although the role of airborne organic nanoparticles cannot be excluded^25^.

The complex mixture of PM$_{2.5}$ includes liquid and solid particles suspended in the ambient air. These particles span in size from a few nanometers to dozens of micrometers, and hugely vary in chemical composition, origins, shape and mixing state. These particles also exhibit complex atmospheric aging processing, and differential toxicities^10,26^. Nonetheless, PM$_{2.5}$ mass alone in the atmosphere is still referred to and used as an air pollution control variable for analysis of human and planetary health. The Global Burden of Disease for ambient air pollution still refers to health risks in one concentration-response (CR) function related to PM$_{2.5}$ mass, which the WHO also uses^8,27^. It is clear that the complexity goes well beyond the currently used PM$_{2.5}$ mass metric, but no robust alternative has been found. The mass concentration of certain components of PM$_{2.5}$, such as Black Carbon (BC), redox-active transition metals and Secondary Organic Aerosol (OA), and the number concentration in certain size-ranges, such as below 100 nm (UFPs), have been proposed^8^. The potential of PM$_{2.5}$ to generate reactive oxygen species (ROS) and produce oxidative stress (the so-called oxidative potential, OP) has also received considerable attention^28–32^. However, our understanding is poor, and PM$_{2.5}$ mass alone still drives health-related policies. It is mandatory that the focus of air pollution studies now shifts to understanding the causal relationship between health effects onset and specific aerosol properties (not just emissions and atmospheric chemistry) and to suggesting health-relevant aerosol exposure guidelines that are planetary in scope^33,34^.

In this study, we show novel data taken during the super-intensive observational periods (SIOPs) of the ’Redox Activity and Health Effects of Atmospheric Primary and Secondary Aerosol’ (RHAPS) experiment, held in the Po Valley in 2021. During the RHAPS experiment, detailed information on physicochemical aerosol properties was measured, together with toxicological markers at the ambient atmospheric PM$_1$ concentrations (2$<PM_1<$ 60 $\upmu$g m$^{-3}$)^35^. During the SIOPs, an additional set of observations was carried out and these are described here for the first time. The aim was to mimic PM$_1$ exposure conditions, representative of human inhalation and deposition, with minimized UFPs sampling artifacts. We used an *in vitro* model of lung bronchial epithelium (BEAS-2B cells) exposed at the air-liquid interface (ALI) to the ambient atmosphere^35–37^. The final scope is to address unresolved scientific questions related to the potency of PM$_1$ and UFPs to induce oxidative and inflammatory responses in the lungs, under ambient atmospheric conditions, with emphasis on low PM$_1$, as well as on the interplay between UFPs and redox-active compounds (the OP). We specifically addressed the interplay between the OP and UFPs, in agreement with most of the literature suggesting that the OP may be one of the many possible drivers of the acute health effects of PM$_{2.5}$^28,30,31^, as well as that UFPs small particle size may result in higher lung deposition and ability to pass into the bloodstream^11,21,38,39^. We combine conventionally used metrics (total mass and number concentrations) to additional parameters of the air mixture, such as the total OP^31^, and the Condensation Sink (CS) as a function of particle size^40–42^. We identified relevant urban aerosol types through the combination of these parameters and then tested these against *in vitro* toxicological outcomes.

A reference model for aerosol exposure onto Air Liquid Interface (ALI) cultures of human bronchial epithelial cells (BEAS-2B) at physiological conditions was used^43–48^. We evaluated cell responses after 24-h exposure. Expression patterns for two reference genes, the Heme oxygenase (*HMOX-1*) and CXC chemokine ligand (*CXCL-8*), indicators of the anti-oxidant defense^37,43,49,50^, and pro-inflammatory^37,51,52^ response pathways respectively, are finally considered for exploring and describing novel unreported associations between aerosol physicochemical properties and adverse health response markers.

## Material and methods

Measurements were carried out during the of the experiment. SIOPs were carried out at the urban background site of Bologna (BO) in the Po Valley , Italy, one of the major air pollution hotspots in Europe.

The entire RHAPS experiment was carried out from January 2021 to July 2021 (we reckon that data may be influenced by the COVID-19 pandemic). Within the RHAPS experiment, SIOPs lasted 16 days. These 16 days were split into four 4-day periods (SIOP1-4). Each SIOP was conducted from Tuesday morning to Saturday morning. The timing was as follows:

- SIOP1) 26 January h 08:00 am / 3 February h 08:00 am;
- SIOP2) 2 February h 08:00 am / 6 February h 08:00 am;
- SIOP3) 16 February h 08:00 am / 20 February h 08:00 am;
- SIOP4) 29 June h 08:00 am / 2 July h 08:00 am.

The winter and summer SIOP periods were planned in advance based on previous data analysis. However, the exact dates of each SIOP were decided week-by-week according to the results of a proper air quality model run every week to forecast weather conditions and selected variables. This subset of variables, including particle mass and number concentration, the black carbon to organic aerosol (BC-to-OA) ratios, and particle diameter, were selected according to our previous knowledge. Basically, we aimed at: (i) catching aerosol accumulation in the atmosphere by having each SIOP last four consecutive stable weather days, starting from a clean day (i.e., good weather possibly following bad weather conditions); (ii) starting SIOPs on Tuesdays and ending on Saturdays to be consistent with traffic emission source paths; (iii) having SIOP representative of source-specific aerosol types, biomass burning, urban aerosol high in traffic emissions, secondary aerosols, and clean conditions.

In addition to the detailed observations of aerosol physicochemical properties carried out during the entire RHAPS experiment, only during the SIOPs cell exposure at the Air Liquid Interface (ALI) and particle-bound ROS were assessed. Methods used to produce the data described in this paper are described in details below. The description of the overall RHAPS experiment can be found in a previous paper^35^ and only a brief summary is provided below. Detailed quality assurance and quality control procedures are described in earlier work^36,37^, and references therein.

### Experimental set-up

During the entire RHAPS experiment, field observations were carried out in the province of Bologna (BO) using a tandem combination of urban background (BO) and rural sites (San Pietro Capofiume, SPC, 30 km NE of BO). Aerosol physicochemical properties in PM$_1$ (chemical components, metals, particle number size distribution, and optical properties) were measured both continuously (time resolution of minutes to hours), and off-line (daily filter collection). Daily filters were also collected for laboratory analysis of oxidative potential and toxicological endpoints.

Oxidative potential (OP) was measured daily (from 8 a.m. to 8 a.m.) both during the SIOPs and during the entire RHAPS experiment. PM$_1$ samples were collected on membrane filters by daily (24h) samplings using low-volume (1.15 m$^3$ h$^{-1}$) sequential samplers. The daily measurements were carried out in parallel, collecting approximately 60 samples at the Bologna and San Pietro Capofiume sites in winter (from 21 January to 18 March 2021) and 35 in summer (from 8 June to 14 July 2021).

Several parallel sampling lines were used at both sites to collect samples for a complete chemical characterization of the aerosol in terms of mass concentration, elements, ions, and carbon components. Daily PM$_1$ samples were collected (from 8 a.m. to 8 a.m.) on 47 mm diameter polytetrafluoroethylene ( PTFE ) filters (Pall R2PJ047, Pall Life Sciences, Ann Arbor, MI, USA) and precoated quartz fiber filters (PallflexTissuquartz 2500 QAO-UP, Pall Life Sciences, Ann Arbor MI, USA). The mass concentration was determined gravimetrically on PTFE filters using a Sartorius microbalance with a sensitivity of 1 $\upmu$g and it ranges from 0.1 to 1.5 $\upmu$g per filter with an average value of 0.5 ± 0.3 $\upmu$g over the entire monitoring period (including both summer and winter).

### Aerosol properties

#### Non-refractory PM$_1$ components

The mass loading and chemical composition of submicron aerosol particles were obtained online by the High-Resolution Time-of-Flight Aerosol Mass Spectrometer (HR-TOF-AMS, Aerodyne Research) [32] at both locations. The HR-TOF-AMS provides measurements of the non-refractory sulfate, nitrate, ammonium, chloride, and organic mass of the submicron particles (NR-PM1). The working principle of the HR-TOF-AMS is described in detail in [32,33,34]. Briefly, during all the campaigns, the HR-TOF-AMS was operating in V ion path modes every 2.5 min. The resolving power [35] of the V-ion mode was about 2000-2200 during all the campaigns. Ionization efficiency (IE) calibrations were performed before and after every campaign and approximately once every two weeks during the campaigns. Filter blank acquisitions during the campaign were performed at least once a day to evaluate the background and correct the gas-phase contribution. All data were analyzed using the standard ToF-AMS analysis software SQUIRREL v1.57 and PIKA v1.16 (D. Sueper, available at: http://www.cires.colorado.edu/jimenez-group/ToFAMSResources/ToFSoftware/index.html, accessed on 1 September 2021) within Igor Pro 6.2.1 (WaveMetrics, Lake Oswego, OR, USA). The HR-TOF-AMS collection efficiency (CE) was calculated based on aerosol composition, according to [36] and confirmed against parallel offline measurements. At both sampling stations, the aerosol was dried to about 35-40% by means of a Nafion drier before sampling with the HR-TOF-AMS.

#### Black carbon

A 7-wavelength (370, 470, 520, 590, 660, 880, and 950 nm) aethalometer (model A33, Magee scientific [46]) provided eBC mass concentration and AAE with 1 min time resolution. According to the instrument manufacturer, the eBC mass concentration from AE33 was obtained from measurements at $\lambda$ = 880 nm with a mass absorption coefficient of 7.77 m$^2$/g The aerosol sampling line was dried to about 20–30% by means of a Nafion drier.

#### Particle number size distribution

The Particle Number Size Distribution (PNSD) was measured by combining a Mobility Particle Size Spectrometer (TROPOS SMPS) equipped with a butanol-based condensation particle counter (CPC, model 3772, TSI Inc., Shoreview, MN, USA) and a commercial aerodynamic particle sizer (APS, TSI). The aerosol sampling line was dried to about 20–30% by means of a Nafion drier.

#### Condensation sink

The aerosol condensation sink (CS) is a commonly used measure of how rapidly molecules condense onto pre-existing aerosols^40,42^. For particles with diameter d$_p$ and particle number size distribution $N_{d_p'}$, the CS was calculated by (Eq. 1):

1 $$\begin{aligned} \ CS= 4 \pi D \int _{d_p min}^{d_p max} \beta _m(d_p) \cdot d_p \cdot N_{d_p} dd_p\ \end{aligned}$$

D is the diffusion coefficient of the condensing vapor (considered to be sulfuric acid, as usually assumed). $\beta _m$ is a transition regime correction defined as a function of the Knudsen number (Kn = 2$\lambda / d_p$):

$$\begin{aligned} \beta _m = \frac{1+Kn}{1+1.677Kn+1.333Kn^2} \end{aligned}$$

In the molecular regime (Kn $>>$1, i.e. particle diameters $>>$ 100 nm), CS is proportional to the square of particle diameter. In the continuum regime (Kn $<<$1, i.e. particle diameters $<<$ 10 nm), CS is proportional to the particle diameter. Our data (8 nm $<d_p<$1000 nm) are in the transitional regime.

The CS (Eq. 1) was integrated upon different particle size ranges ($d_pmin$–$d_pmax$): $d_p<$20 nm (in the nucleation mode, here indicated as CS$_{8-20}$), 20$<d_p<$40 nm (in the Aitken mode, here indicated as CS$_{20-40}$), 40$<d_p<$100 nm (in the soot-mode, here indicated as CS$_{40-100}$), 100$<d_p<$200 nm (in the condensation mode, here indicated as CS$_{100-200}$), 200$<d_p<$900 nm (in the larger accumulation mode, here indicated as CS$_{200-900}$).

#### Source apportionment of PNSD

The particle number size distribution (PNSD) was statistically analyzed to identify major aerosol types. We already described the methodology in previous papers^53,54^. Briefly, to identify statistically independent factors in the urban aerosol, we conducted principal component analysis (PCA) encompassing aerosol particle number size distributions. The resulting PCs, outstanding with respect to their temporal persistence, could be associated with aerosol particle modes^53^. Then, we combined PCA results with a clustering analysis to categorize the collected aerosol size distributions into main categories, and hence major aerosol types. The complete results of this statistical analysis can be found in the SI, Figs. S8, S9.

The PCA analysis shows three major components explaining 87% of the temporal variance; these were similarly extracted by the cluster analysis. To describe these in terms of comparable variables relevant for modeling and process analysis, we fitted the modes identified by both the principal component and the cluster analysis with the multi-log-normal distribution function^55^ and calculated the three parameters characterizing relevant individual lognormal modes, i.e. the mode number concentration (N), the geometric variance $\sigma$, and geometric mean diameter $\upmu$. There is only one component (PC2, Cluster 2, the second in terms of variance explained, 26% ) showing an almost unimodal particle number size distribution with $\upmu$ below 20 nm ($\upmu$=18 ± 2 nm). It shows a very small second mode at $\upmu$= 48 ± 18 nm, and no accumulation mode. In the wintertime, it peaks at the rush hours of the weekdays and doesn’t occur on the weekend. In the summertime, it shows an additional midday peak. We explain this component by traffic emissions and photochemically driven secondary particle formation associated to traffic emissions, consistent with our previous frequent observations^53^ in urban areas of an aerosol type with monomodal number size distribution in the 3–20 nm particle size range occurring at midday/early afternoon after photonucleation occurs and following traffic rush hours.

We note that the particle size range identified by the dry electrical mobility diameter smaller than 20 nm (in fact, 8–20 nm, based on the lowest cut-off of the instrument) almost uniquely indicates this aerosol type. Accordingly, we considered the number concentration of particles in the 8-20 nm size range (N$_{8-20}$) as a first indicator for traffic-related photonucleation mode particles.

Although the statistical analysis shows a mode centered at $\upmu$=18 ± 2 nm, we recall that our data are constrained by the lowest size cutoff at 8 nm of measurements: the lognormal fitting suggests an additional nucleation mode centered between 1 and 10 nm. However, the smallest size cut of the instrument doesn’t allow us to track exactly a possible nucleation event, and we consider these particles as shortly aged in the atmosphere.

#### Metals

Water-soluble and insoluble metal concentrations for Al, As, Ba, Bi, Cd, Ce, Co, Cr, Cs, Cu, Fe, Ga, K, La, Li, Mn, Mo, Na, Pb, Rb, Sb, Sn, Ti, Tl, U, V, W, Zn, and Zr, were obtained from PTFE filters collected every 24 hours (from 8:00 a.m to 8 a.m) at the urban background site of BO in PM$_1$ samples, analyzed by ICP-MS.

#### PM$_1$ mass concentration

PM$_1$ mass concentration with 5 min time resolution was constructed from the PNSD data, according to the procedure described elsewhere^36^. In short, PM$_1$ was calculated from the particle volume size distribution under the hypothesis of spherical particles, and a size-dependent particle density varying from 1.25 to 1.5 g /cm$^3$ was assumed. The daily PM$_1$ from SMPS was then validated according to the daily PM$_1$ measured through the reference procedure, the goodness of fit being R$^2$ = 0.99.

### Biological responses

#### Cell culture

The in-vitro test system consisted of an immortalized epithelial cell line derived from normal human bronchial cells (BEAS-2B cells 95102433, ECACC, Salisbury, UK). BEAS-2B cells, currently used for air pollution toxicological studies, retained a large representativeness of the normal bronchiolar epithelial cells^43,44,47,48,56^.

The human bronchial epithelial cells were maintained in LHC-9 medium at 37$^\circ \,\hbox {C}$ with 5% of CO$_2$, and split every 3 days. 72 h before exposure BEAS-2B were seeded on collagen-coated inserts (12 wells multiplate Teflon transwell inserts with 0.4 $\upmu$m pores, collagen-coated Corning, NY, USA) at a density of 40x10$^3$ cells/insert and let to grow. The inserts were then transferred to the site of exposure and 24 h before starting the first experiments, the medium at the apical side of the transwell was removed to let the cells at the air-liquid interface and promote cell differentiation. The exposure of the lung model was, therefore, performed with cells constituting a continuing layer, approximately 100% confluence, according to the physiological condition of lung epithelia.

#### Exposure at the air liquid interface

The exposure procedure was performed according to^37^ with slight modification. Briefly, six inserts were transferred into the CULTEX$\circledR$ RFS Compact module and the basal side of each chamber was filled with 4 mL of LHC-9 medium (Gibco, Life Technologies, Monza, Italy) just before the beginning of the exposure.

The exposure technology selected is reported to allow for an optimal and uniform distribution of particles of different dimensions among the different inserts with minimal losses^57^.

Cells were exposed at ALI to the native atmosphere (with a cutting cyclone to 1 $\upmu$m of aerodynamic diameter ) or to filtered environmental air (air was first bubbled in sterile water, then passed through a charcoal filter to remove remaining volatile compounds and then through an absolute filter to remove the particulate phase) for 24 h from 8 AM to 7:59 AM of the subsequent day. The temperature of the exposure chamber was maintained at 37$^\circ \hbox {C}$. It is well known that this generates a water vapor phase that keeps the exposure chamber a 100% as expected in the lung. During each week of exposure, four independent experiments were performed. At the end of each exposure, each insert was visually scored for clear signs of sufferance by a reverted microscope (Zeiss Axiovert 100, Germany).

Among the six exposed inserts, one control and one exposed insert randomly chosen and were manipulated to collect mRNA and to assess the differential expression of the following genes (HMOX-1 and CXCL-8, discussed here and relevant to assess the oxidative and pro-inflammatory potential of airborne PM^58,59^, and Gadd45$\alpha$, NQO1, ATM relevant for DNA damaging pathways, also the release of the interleukin IL-8 was assessed in the culture medium) one control and one exposed for DNA damage analysis (by Comet Assay), and the two-remaining insert for protein extraction (for possible proteomic characterization). Variation in the gene expression was evaluated in triplicate and both considering down-regulation and up-regulation of the selected genes according to^60^. During each week of exposure, three additional inserts were kept in the local incubator and considered as additional references to the biological response of cells exposed through the ALI module.

Exposure doses of cells were calculated according to^37^ . Briefly, SMPS data (as reported in^35^ and in the section above) were used to define the mass size distribution according to the procedure described elsewhere^36^. The deposition of particles, according to the size and density was calculated considering impaction and random sedimentation^61^. Previous studies have shown that the deposition efficiency of micro and nanoparticles have a higher stability of the systems in terms of even distribution of the mass deposited and a high efficiency of deposition at different size ranges^57^.

#### Limitations for cell exposure at the ALI

The in-vitro model used during our experiments, although largely accepted^48,62^ as relevant for assessing airborne contaminants’ hazards and toxicity, still presents some limitations. The model is representative of the bronchial space in proximity to the alveolar space, and far from the histological complexity of the upper respiratory tract with cylindrical or pseudostratified epithelial cells. Moreover, lung homeostasis relies on the interplay of different cell types (such as mucus or surfactant-producing cells, macrophages, and other immune cells) and the possible combined effect of different cell types should be considered in the future. The interaction of particles with lung epithelia is driven, among other factors, by the presence of the lung fluid which can activate or de-activate particle bounded chemicals^63^ . In our study, we did not add a lung fluid-mimicking solution. The lack of lung fluid in our model may have affected the responses of the cells but the direction of this modification (increased or decreased responses) could not be defined considering the actual understanding of the interaction between PM and the lung fluid and should be investigated in detail in future research. The limitation of the biological replicates is in our opinion a possible confounding factor in our experiments. The robustness of the exposure module, in terms of even distribution of the sampled particles, is high enough to avoid intra-experiment bias, but a wider battery of biological replicates should be envisaged in future experiments to properly account for the biological variability of the intra-experiments results. Our previous experiments^37^ showed however minor variation between intra-experiments biological replicates.

#### Biological endpoints

The expression of oxidative (*HMOX-1* ) and inflammatory (CXCL-8) genes was quantified after 24 h of exposure. These genes were presented in a previous paper^35^.

Among the inflammatory mediators, whose release may be triggered by the inhalation of particulate matter, the available literature suggests that CXC chemokine ligand (CXCL)8 is a highly important one^37,51,52^.

The Heme oxygenase (*HMOX-1* ) was selected as a significant biomarker of exposure, being related to the antioxidant response pathway. Indeed, *HMOX-1* has been often used in literature, known to be highly responsive to oxidative stress and to play a protective role in response to several stressors^43,49,50,64^. A specific discussion for gene selection is beyond the scope of this paper. The following forward and reverse primers were selected to analyze the expression of the selected genes: *HMOX-1* forward CAACAAAGTGCAAGATTCTGCC and reverse TGGCATAAAGCCCTACAGCA; *CXCL-8* forward GAAGTTTTTGAAGAGGGCTGAGA and reverse CACTGGCATCTTCACTGATTCT.

Significant differences were observed in the expression of the two genes during the different exposure days with a general higher expression in winter than in summer^35^.

### Aerosol oxidative potential

#### OP

During the RHAPS experiment the aerosol oxidative potential was measured by the most used methods for OP^30–32,65^ : dithiothreitol (DTT), ascorbic acid (AA), and the 2,7-dichlorofluorescin (DCFH) assays . The DTT was used on both quartz and Teflon filters. The aim of these acellular assays is to provide a proxy for the oxidative capacity of PM samples. Among these methods, only the “total DTT” on quartz filters^31^ showed statistical association with HMOX-1 and CXCL-8 gene expression (Fig. S1 in the SI Appendix). We therefore refer only to “total DTT” OP^31^ in the current work. We refer to the SI Appendix, paragraph 1, for a discussion on the limitations and strengths of the different assays.

A sampling line based on quartz fiber filters was devoted to the OP determination on the soluble and total fraction of PM$_1$ by the dithiothreitol (DTT) assay. The adopted procedures are those by^31^ for the total OP and for WSOP by^66,67^. Filter portions were extracted in deionized water by gentle shaking (30 min). The quartz fiber filter aliquots were not removed from the extraction solution after the end of the extraction procedure, and they were kept in the primary vial while performing the DTT assay. This was intended to allow both soluble (in the extract) and insoluble (attached to the filter) aerosol components to react with the DTT.

The mass of PM collected on the filters was largely sufficient throughout the monitoring period to obtain OP values above the detection limits^35^ for most of the DTT measurements (as well as for AA and DCFH measurements). The daily mass of PM collected on filter membranes varied from 0.1 to 1.5 mg per filter with an average value of 0.5 ± 0.3 mg over the entire monitoring period (including both summer and winter), the amount of dust collected was, therefore, sufficient to obtain measurements of OP above the detection limits for more than 90% of the analytical determinations.

#### Particle-bound ROS

An online (2-h time resolution) cell-free system was used for the assessment of the particle-bound ROS^30,68^. A particle-into-liquid sampler (PILS) allowed for the continuous aerosol collection of a diluted solution of soluble species with suspended insoluble particles.

Ambient air was sampled at the flow rate of 15 L min$^{-1}$. The sampling line was equipped with a PM_1_ inlet and a denuder line to keep acid and basic gasses out of the sample. Samples were analyzed through the 2,7 dichlorofluorescein (DCFH) assay. Data were converted to units of nmol H$_2$O$_2$ equivalents. Particle-bound ROS concentrations are reported as nmol H$_2$O$_2$ equivalents during the 2-h sampling intervals normalized to the volume of air sampled by the instrument during the 2-h interval, presented here as volume-normalized levels (in units of nmol H$_2$O$_2$ m$^{-3}$ of air). Also, these were normalized to the PM1 mass ($\upmu$g of PM_1_) measured during the same 2-h period ($\upmu$g m$^{-3}$ multiplied by the volume in m$^{-3}$ of air sampled by the instrument during the same 2 h) and presented here as mass-normalized values (in units of nmol H$_2$O$_2$ $\upmu$g$^{-1}$ of PM_1_ mass).

We reckon that this technique does not guarantee the complete recovery of small and hydrophobic particles because particle growth is achieved through water condensation.

### Statistical analyses

Statistical analyses were performed using R programming version 3.2.4 (http://www.R-project.org; Te R Foundation for Statistical Computing, Vienna, Austria).

## Results

**Figure 1:**
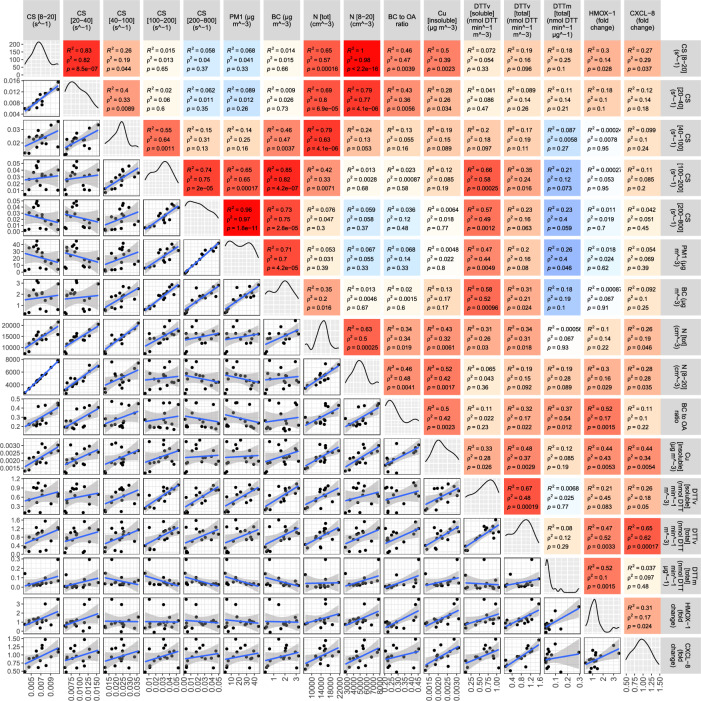
Statistics for comparison between atmospheric aerosol properties and related pro-inflammatory and oxidative endpoints. Paired scatterplot matrix shows 24-h data for variables related to bulk aerosol (PM1, and BC mass concentration, and total number concentration, N$_{tot}$); the Condensation Sink (CS) as a function of particle size, in the nucleation, Aitken, soot, condensation and larger accumulation mode (CS$_{8-20}$, CS$_{20-40}$,CS$_{40-100}$, CS$_{100-200}$, CS$_{200-900}$, see “Method” Section); aerosol type (number concentration of nucleation mode particles, N$_{8-20}$, BC-to-OA ratio, and insoluble Copper mass concentration (Cu)); both water-soluble and total aerosol oxidative potential (DTT activity of PM$_1$ samples); gene expression for oxidative stress (fold change for *HMOX-1* and inflammation (fold change for *CXCL-8*). Statistical correlation values is shown in the box as Pearson correlation (R$^2$), and Spearman correlation ($\rho ^2$), with the relative significance level (*p*-value). The color gradient is proportional to the Pearson correlation coefficient, R (red for positive R, blue for negative R). The number of points (n) to calculate correlations is 16, except for DCFH where n = 8. Created in R-Studio version 2022.12.0 using ggpairs function from the GGally package version 2.1.2.

Figure 1 shows the scatter-plot matrix and related correlations between a selected subset of atmospheric aerosol properties and toxicological markers of the inflammatory and oxidative response. The entire list of correlations can be found in SI Appendix, Figs. S1–S6 and Table S2, including water-soluble and total OP by different assays (dithiothreitol (DTT), ascorbic acid (AA), 2,7-dichlorofluorescin (DCFH)), mass concentration of insoluble and water-soluble metals (Al, As, Ba, Bi, Cd, Ce, Co, Cr, Cs, Cu, Fe, Ga, K, La, Li, Mn, Mo, Na, Pb, Rb, Sb, Sn, Ti, Tl, U, V, W, Zn, and Zr), non-refractory PM$_1$ components (nitrates, ammonium, sulfates, chloride and organic aerosol primary and secondary components derived from aerosol mass spectrometric (AMS) measurements), Poly-Aromatic Hydrocarbons (PAH), particle number size distributions for diameters ranging from 8 nm to 1000 nm, and gaseous compounds (ozone and NO$_2$). No correlation was found between control gene expression and Ozone and NO$_2$ concentration (SI Appendix, Table S2). Among all data analyzed, only variables reported in Fig. 1 showed statistically significant associations with the biological responses for oxidative stress and inflammation (Pearson’s and Spearman’s correlation coefficients (R$^2$, and $\rho ^2$, respectively) are given with relevant significance levels (*p*-value)). Columns 15-16 of Fig. 1 indicate the fold change in gene expression patterns for *HMOX-1*^37,43,49,50^, and *CXCL-8*^37,51,52^, respectively. Columns from 6 to 8 show three emerging metrics for monitoring, PM$_1$ and BC mass concentration, and the total number concentration (N$_t$).

Columns from 1 to 5 show the Condensation Sink (CS)^40–42^ as a function of particle size, in the nucleation (8–20 nm), Aitken (20–40 nm), soot (40–100 nm), condensation (100–200 nm), and larger accumulation mode (200–900 nm), i.e. CS$_{8-20}$, CS$_{20-40}$,CS$_{40-100}$, CS$_{100-200}$, CS$_{200-900}$. The CS is a well-known indicator of how rapidly a molecule condenses on preexisting aerosols, an important factor controlling new particle formation events, also in urban environments^40,41^. The CS increases with increasing particle diameter and number concentration (Eq. 1), and is higher, especially for particles in the accumulation mode size range. These particles cause most of the PM$_1$ mass, owing to the large correlation between CS$_{200-900}$ and PM$_1$. There is a positive statistically correlation between CS$_{8-20}$ and *HMOX-1* and *CXCL-8* gene expression. Figure 1 shows non-linear CR functions among both PM$_1$ and BC mass concentrations and both *CXCL-8* and *HMOX-1* gene expression. For N$_t$, there is a positive correlation with *CXCL-8*, not statistically significant with *HMOX-1* (*p*-value>0.2). Columns from 9 to 11 show three parameters, interrelated to each other: BC-to-OA ratio, insoluble copper mass concentration (Cu), and number concentration of particles in the size range 8–20 nm (N$_{8-20}$). The BC-to-OA ratio has already been used in laboratory studies to separate fresh (high BC-to-OA) from aged (low BC-to-OA) combustion emissions^69,70^, and in the ambient atmosphere to separate fresh from aged aerosols^71^. There is a positive significant correlation between BC-to-OA and *HMOX-1*, but not statistically significant with *CXCL-8* ($p>$ 0.2). The N$_{8-20}$ shows a positive correlation with both *HMOX-1* and *CXCL-8* (*p*-value <0.05). This is not the case for the number concentrations of larger particles, as observed in the size ranges of 40–100 nm, 100–200 nm, and 200–900 nm (SI Appendix, Figs. S4, S6). These size ranges resulted from statistical analysis of particle number size distributions (PNSDs) (“Materials and methods” section), indicating N$_{8-20}$ to proxy an aerosol type with monomodal PNSD in the nucleation mode, occurring essentially at the daytime of the working days after the traffic rush hours. The insoluble Cu, which is known as a redox-active component in the fine particulate matter and in nanoparticles^72–75^, shows a statistically significant positive relationship with both *HMOX-1* and *CXCL-8* gene expression. No similar relation was found with all the other metals here measured (Si Appendix, Figs. S2, S3). There is a correlation between insoluble Cu mass concentration and N$_{8-20}$ and CS$_{8-20}$. Columns 12–14 of Fig. 1 show the Oxidative potential (OP). The OP is expressed as total and water-soluble DTT activity (nmol DTT min$^{-1}$ m$^{-3}$) of PM$_1$ sample (procedures by^31^ for the total OP, and by^66,67^ for WSOP, details in “Methods” section). We show also the total intrinsic DTT activity (DTTm in nmol DTT min$^{-1}$ $\upmu g^{-1}$). The strongest significant positive correlation for *HMOX-1* and *CXCL-8* is observed with the total DTT (on a per volume basis, DTTv), with lower correlations with the water-soluble DTT. No similar association was found with the other OP assays observed in this study (SI Appendix, Fig. S1). The total DTT correlates with insoluble Cu, BC-to-OA, particle number concentration (more than with particle mass concentration), and the CS in both the accumulation and nucleation mode particles, while no similar relation with UFPs appears for the water-soluble DTT.

**Figure 2:**
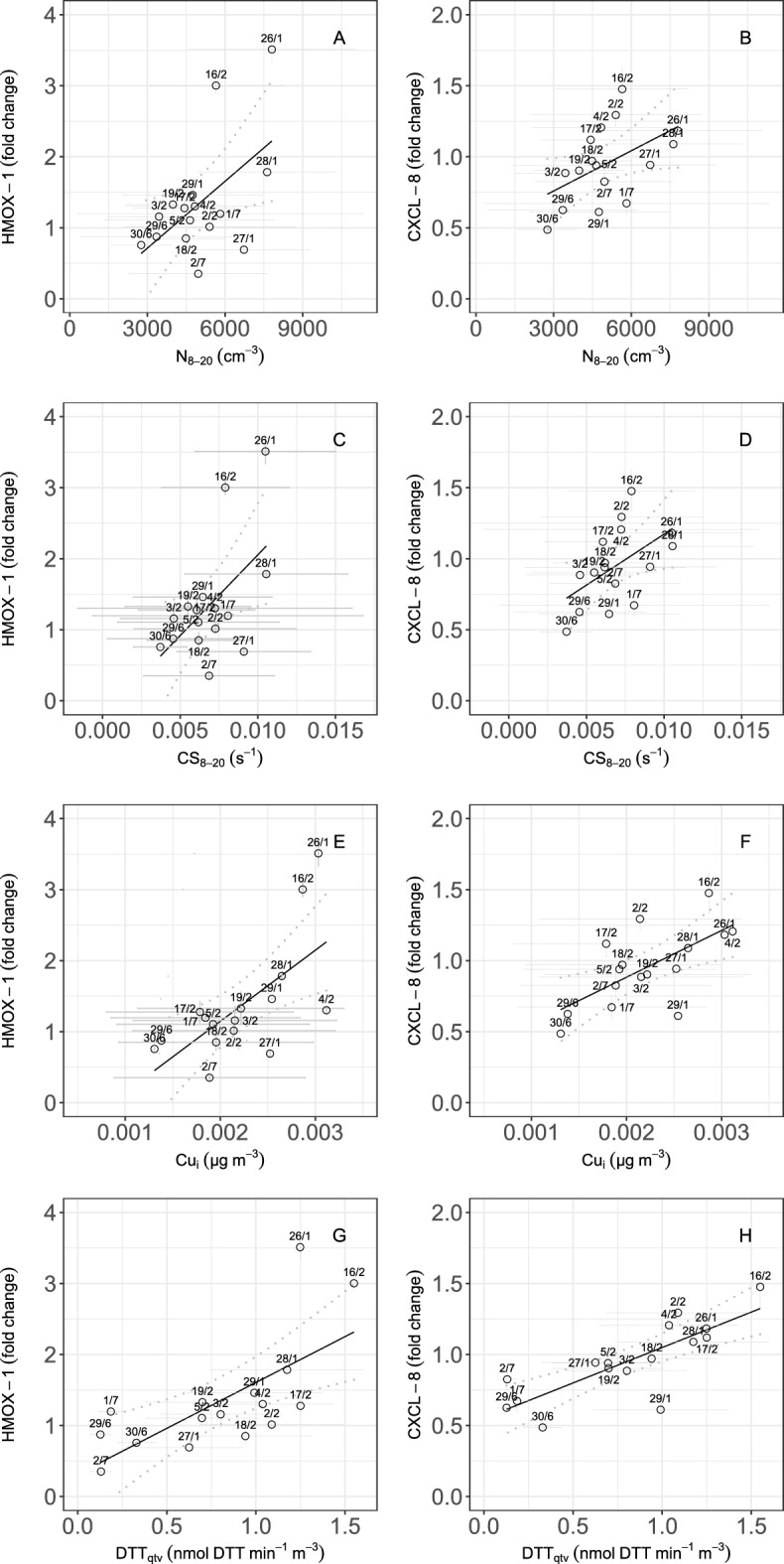
Relationships between atmospheric aerosol properties and oxidative capacity, and pro-inflammatory and oxidative markers. The number concentration of nanoparticles N$_{8-20}$ (panels **A**, **B**), Condensation Sink of nanoparticles CS$_{8-20}$ (panels **C**, **D**), insoluble Copper (Cu) mass concentration (panels **E**, **F**), and (**G**, **H**) total oxidative potential (DTT activity of PM$_1$ samples) is presented against the fold change (f.c.) in gene expression for *HMOX-1* (oxidative stress) and *CXCL-8* (inflammation). Data points are indicated with standard deviation error bars. Linear regression lines are indicated with a 95% confidence interval (dashed ribbons), and relevant date of measurements (dd/m).

Higher R$^2$ in Fig. 1 contemporarily for both *HMOX-1* and *CXCL-8* were found for the total DTT activity, on a per volume basis (R$^2$ of approx. 0.5 with *HMOX-1* and 0.6 with *CXCL-8*) and insoluble Cu (R$^2$ of approx.0.4), and the CS and number concentration of nucleation mode particles (R$^2$ of approx.0.3). In Fig. 2 we show the related scatterplots (*HMOX-1* and *CXCL-8* against the number concentrations and CS of nucleation mode particles (panels A-B, and panels C-D) and the insoluble Copper (panels E-F) and total OP as total DTT activity (panels G-H)), with the indication of 95% confidence interval, standard deviation error bars, and the date of measurements (dd/m). The stronger correlation with *HMOX-1* and *CXCL-8* found for the total DTT than for the insoluble Cu points to contributions to OP from chemical species other than transition metals and probably accounted for by (unspeciated) organic compounds. Overall, the positive correlations between *HMOX-1* and *CXCL-8* with the microphysical and chemical aerosol properties are only moderately strong, which is expected because the actual mechanisms leading to the biological response are probably influenced by combinations of factors. As a first attempt to probe possible synergic effects from multiple aerosol properties, we performed a simple multi-linear correlation analysis (SI Appendix, Fig. S9). This shows that the highest correlation factor, with approx. R$^2$ = 0.7 (70% of explained variance) for both *HMOX-1* and *CXCL-8*, is associated with a linear combination of the total OP (as total DTT) and the condensation sink (CS), with a negative coefficient for the CS if in the accumulation mode (CS$_{200-800}$). This negative contribution for CS$_{200-800}$ appears in Fig. 1, showing that while the per-volume DTT activity increases with both the CS$_{8-20}$ and CS$_{200-800}$, the per-mass DTT exhibits a negative correlation with CS$_{200-800}$.

**Figure 3:**
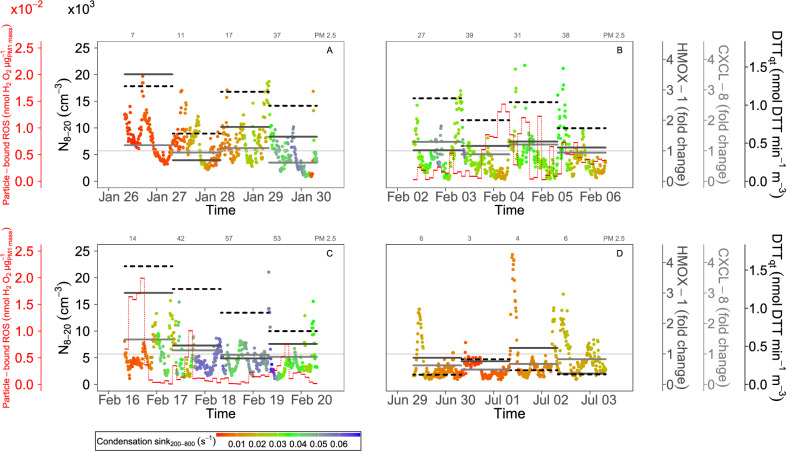
Temporal variability of atmospheric aerosol properties relevant for the pro-inflammatory and oxidative endpoints. The 10-min time series of the number concentration of nanoparticles (N$_{8-20}$) is indicated on the left primary y-axis. Data points are colored by the condensation sink of accumulation mode particles (CS$_{200-800}$). The 24-h data for *HMOX*-1 (black line) and *CXCL*-8 (grey line) f.c. gene expression is indicated on the secondary right y axes. The 24-h data of the aerosol oxidative potential (DTT activity in PM$_1$ quartz samples) is indicated (dotted black line) on the secondary right y-axis. The 2-h data of particle-bound ROS (as PM_1_ mass-normalized values) is displayed on the secondary left axis (dotted red line). The 24-h values of PM_2.5_ mass concentration ($\upmu g \cdot m^{-3}$) measured by the local environmental agency are indicated at the top of the plots. Note the parallel gene up-regulation on: Jan 26 (*HMOX*-1 f.c.=3.51±0.18 with $p<$0.01, *CXCL*-8 f.c.=1.18±0.08 but $p>$ 0.05); Feb 16 (*HMOX*-1 f.c.=3.00±0.10 with $p<$ 0.01, *CXCL*-8 f.c.=1.47±0.15 with $p<$0.05); Jan 28 (*HMOX*-1 f.c.=1.78±0.05 with $p<$ 0.01, *CXCL*-8 f.c.=1.09±0.01 with $p<$0.01); Feb 04 (*HMOX*-1 f.c.=1.30±0.06 with $p<$ 0.01, *CXCL*-8 f.c.=1.20±0.03 with $p<$ 0.01). On Feb 03, there is data missing: (i) from h 09:06 to h 12:45 cells’ exposure was discontinued due to the activation of a diesel generator, and the line is thinner; (ii) from h 15:00 to 20:00 the OA data is missing and the marker color is grey.

In Fig. 3 we explore the mechanisms linking aerosol microphysics and OP to *HMOX-1* and *CXCL-8* at timescales shorter then 24 h. We show a 10-min time series of N$_{8-20}$ (primary y-axis) together with the CS of the accumulation mode particles (marker color). These are plotted against 24-h data of *HMOX-1* and *CXCL-8* (black and grey lines, secondary y-axes) and the total OP (as DTT activity, black dotted lines). In this study, the DTT assay was applied only to time-integrated (24 h) samples, but we make use of the fast (2-h integrated) measurements (using the DCFH assay, details in “Methods” section), a measure for particle-bound ROS concentration^30^, to gain further insight into the relationship involving ROS. These (when available) are also displayed as red dotted lines (as mass-normalized particle-bound ROS concentration, positively associated with *HMOX-1*, SI Appendix, Fig. S10; consistent patterns for the ROS concentration on a per volume basis are shown in SI Appendix, Fig. S11). Daily reference values for PM$_{2.5}$ are indicated in the top, while PM$_1$ is indicated in SI Appendix, Table S1. We can observe that the days with low PM$_{2.5}$ concentrations and high gene expressions (26 Jan and 16 Feb, cf. Fig. 2) are characterized by high N$_{8-20}$ and low CS$_{200-800}$, and high total DTT. Conversely, days with high PM$_{2.5}$ concentrations and total DTT (28 Jan, 29 Jan, 17 Feb, 18 Feb) but high CS$_{200-800}$, show low *HMOX-1* and *CXCL-8*. As well, days with high N$_{8-20}$ but low total DTT (in the summer period, 29 June, 1 and 2 July) are characterized by low gene expression. Another feature that can be observed in Fig. 3 is that the days with high total DTT activity are characterized also by high particle-bound ROS concentration, with the latter showing peaks at times of the day when CS$_{200-800}$ is minimum. On 16 Feb, in particular, the mass-normalized particle-bound ROS concentrations reached the highest values of the campaign in the central hours of the day, when CS$_{200-800}$ was low. Similar but smaller features were found around midday on 17 and 19 Feb, while the period of 2 to 6 Feb shows larger variability, but with a peak of the particle-bound ROS in the morning of 2 Feb whit CS$_{200-800}$ again very small.

## Discussion

In this study, we show novel data based on a methodology recently developed^36,37,54^ to reduce artifacts for exposure of lung epithelial cells to the urban UFP-rich aerosol in the ambient air. This allows assessing also exposure to low doses of PM$_1$, for which there is a paucity of data in the literature^7,8^. Oxidative stress and inflammation gene expression (*HMOX-1* and *CXCL8*) are assessed in BEAS-2B cells exposed at the ALI to the urban aerosol. Our data show higher *HMOX-1* and *CXCL8* for higher levels of the total OP (traced by total = soluble and insoluble DTT activity) with a high content of insoluble Cu, coupled to higher nanoparticle levels (N$_{8-20}$ and CS$_{8-20}$) and low CS of accumulation mode particles ( CS$_{200-800}$).

**Figure 4:**
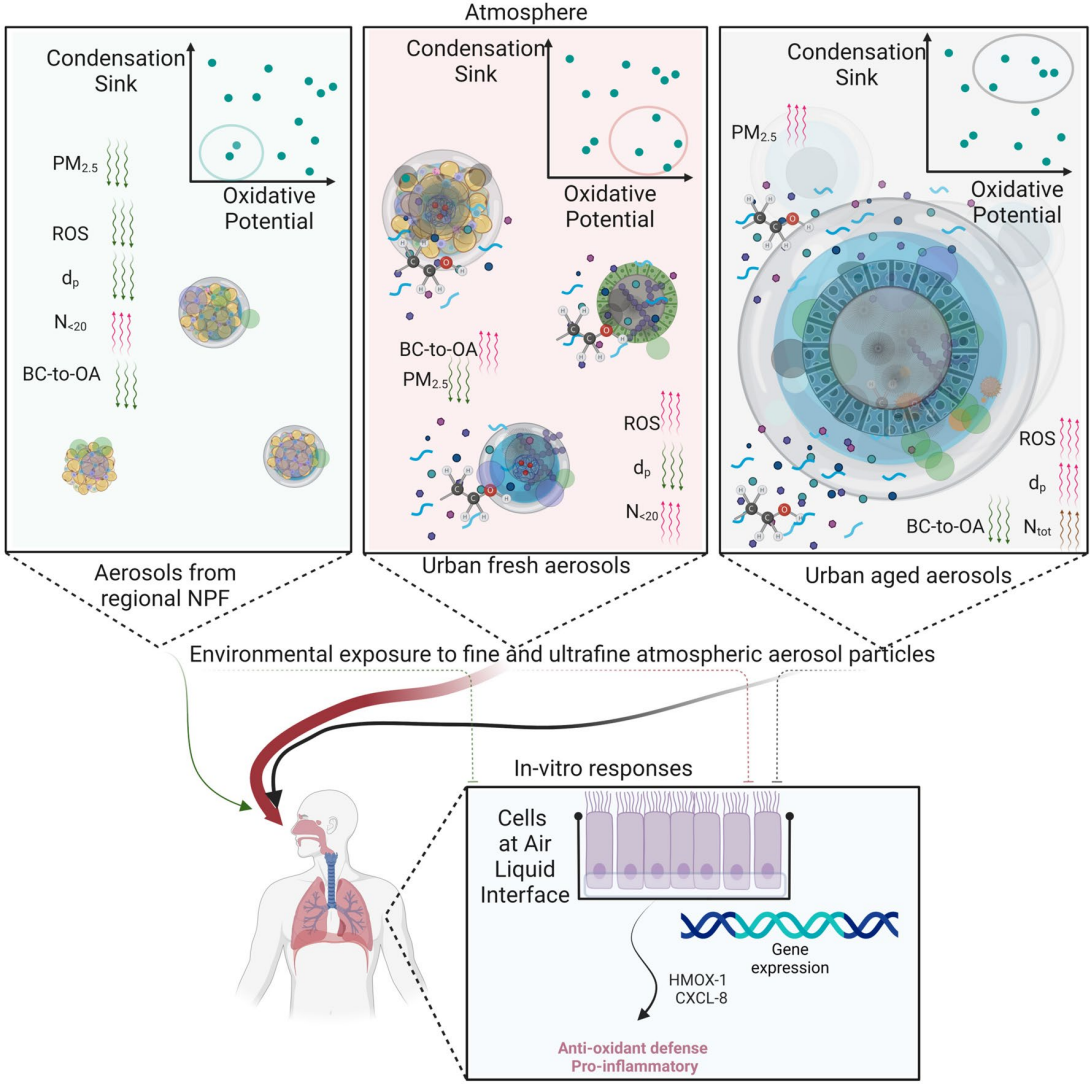
Hypothesis on the relationships between atmospheric aerosols and biological endpoints. Considering the urban atmosphere, we hypothesize three major aerosol types: the aerosol from regional new particle formation (NPF) events, as shown in the top left side; the urban fresh aerosol (high BC-to-OA), as shown in the top center side; and the urban aged aerosols (low BC-to-OA), as shown in the top right side. These are parametrized by the combination of Condensation sink and the Oxidative Potential, in the top right side of each box. Also, the expected values of the Reactive Oxygen Species (ROS), particle diameter (d$_p$), and number concentration of nucleation mode particles (N$_{20}$), total particles (N$_{tot}$) and PM$_{2.5}$ are indicated. In-vitro responses of lung epithelial cells (BEAS-2B) exposed at the air-liquid interface (ALI) directly in the environment (mimicking the human exposure by inhalation) to this aerosol are analyzed by the anti-oxidant defense (HMOX-1) and anti-inflammatory (CXCL-8) gene expression in BEAS-2B cells directly exposed at the Air Liquid Interface in the atmosphere. Created with BioRender.com.

We offer possible explanations for these findings in Fig. 4. We propose the interplay between the CS and the OP as a conceptual model to assess oxidative stress and inflammation. In Fig. 4, the *urban fresh aerosol* features high redox-active components and particle-bound ROS (total OP) enriched in nanoparticles with low amounts of larger preexisting accumulation mode particles (low CS$_{200-800}$). This may occur in urban areas at the rush hour on a winter weekday, after bad weather conditions. Bad weather conditions, with rain and strong winds, would favor a lowering of the accumulation mode particles, and hence a lowering of the PM$_1$ mass concentration. At rush hour on a winter weekday, traffic emissions are typically abundant in urban areas. These conditions, being coupled together, would favor the increase of high redox-active components and particle-bound ROS enriched in nanoparticles, with low PM$_1$. The low value of the CS$_{200-800}$ is indicative of an atmospheric aerosol where condensable compounds (including ROS) do not sink rapidly on preexisting accumulation mode particles, and are rather scavenged by ultrafine particles.

We provide a possible explanation of how the CS and the concentration of redox-active compounds (like insoluble copper) and nanoparticles may interact to affect oxidative stress and inflammations. Once the condensed redox-active organic compounds become enriched in nanoparticles that already contain reactive compounds like trace metals (Cu), they can exert a stronger toxicological response, as compared to their same content in different forms (e.g., same redox-active compounds but in accumulation mode particles, associated with higher CS ). This mechanism would be consistent with the Trojan-horse mechanism already reported for nanoparticles associated with heavy metal constituents^16,22,23^, that were found to have higher toxic responses as compared to exposure to the same metal mass in an ionic form and/or in macroscopic particles. Relevant processes may include increased nanoparticle mobility, lung deposition efficiency, and ability of nanoparticles to cross cell membranes^16,22,23^.

We note that the DTT activity can be augmented by the condensation of reactive species from the gas phase (such as quinones and other secondary organic compounds). Higher CS values translate to higher PM$_1$ values, and therefore higher values of the volume-based OP^32^ . However, in our experiment, a higher intrinsic total DTT activity was found for nucleation mode particles (Fig. 1). These nanoparticles have indeed a higher deposition efficiency in the lungs^38^^21^^39^^11^. Therefore, condensation occurring upon nanoparticles (at low PM$_1$) might lead to higher gene expression values for *HMOX-1* and *CXCL-8*. Such an effect contributes to explaining why a decrease in PM$_1$ air concentrations might not translate into a reduction of toxicity. The *urban fresh aerosol* represents this aerosol type with higher numbers of these specific nanoparticles with high total OP, associated with higher inflammatory and oxidative biomarkers. For comparison, we indicate in Fig. 4 an *urban aged aerosol* (lower BC-to-OA) and larger particle diameters (larger total CS). This would represent a condition typically occurring in urban areas during winter “stable weather” days. Under these conditions, aerosol typically accumulates in the urban atmosphere, and hence particle aging is favored. As a third exemplary case, we indicate nucleation mode particles, which are low in the CS$_{200-800}$, but have a lower OP, sourced by processes other than combustion-related ones. This is a condition typically occurring in the summer season at rural locations, where regional new particle formation may occur.

This is discussed in the following paragraphs in coherence with established literature, with some implications in the use of OP as control variables in health studies, and of PM$_1$ as an indicator of oxidative stress and inflammation. Although novel data are presented in our study, results are consistent with recent literature^7,8,12,13,16,22,23,26,28,43,44,49,50,72–84^. Epidemiological studies have already called for possible health effects at low doses, although toxicological studies at low PM$_1$ are scarce^7,8,12,13,76,77^. Laboratory/modeling experiments have already indicated a number of aerosol properties connected to oxidative stress and inflammation, including the OP^28^ and UFPs^8^, although experimental data directly in the atmosphere at low PM$_1$ and compared to gene expression patterns are scarce. Consistency may be found between our results and this body of literature indicating as key factors the decreasing particle size^43,50,78^, certain metals - especially copper - enriched in nanoparticles^16,22,23,72–75^, the bulk particles per sè^43,49,50^, specific molecules over particle surface^26,44^ and/or into the bulk in association with a specific phase state^79–82^, certain gaseous compounds around the particles^44,82,83^, anthropogenic sources^78,84^, organic coatings on the BC particles and atmospheric aging of the aerosol^78^, and/or the combination of all of these factors (summarized in SI Appendix, Table S3).

### Different types of nanoparticles have different toxicities

Consistent with previous studies^38,39,43,50,78^, our data point at the relevance of particle size in the generation of inflammatory and oxidative responses. The role of particle size is emphasized in this study by the methodology adopted. We measured UFPs properties online (no filter collection) and associated these to toxicological markers measured through an exposure module where not all particle sizes penetrate with the same efficiency (mimicking the lung): not all particle sizes, but only those of particles deposited at the ALI upon cells contribute to the resulting dose and the toxicological marker^37–39,43,78^. On the other side, data also highlight that particle size alone is not sufficient to characterize these toxicological markers. That is to say that not all nanoparticles should have the same toxicity.

Complex trends for N$_{8-20}$ were observed during our experiment, with higher number concentrations during traffic rush hours, as well as at other hours of the day and during weekends, and during likely regional NPF events. Clearly, not all particles with a diameter less than 20 nm belong to the *urban fresh aerosol*. We found (Fig. 3) higher *HMOX-1* and *CXCL8* only when N$_{8-20}$ is associated with high total OP (DTT activity) with both low CS$_{200-900}$ and high insoluble Cu (the features of the *urban fresh aerosol* in Fig. 4). These nucleation mode particles, observed for example on 26/01, but also on 16/02 (Fig. 3, and SI Appendix, Fig. S7, S8) persisted for a few hours but didn’t grow to form the typical banana-shape, the lack of which is usually a signature of particle formation processes that are not regional New Particle Formation, NPF^40^. Particle growth above 30–40 nm is less common in urban-type nucleation events, constrained in the late afternoon by the availability of solar radiation intensity and by the increased urban CS$_{200-900}$ (due to traffic-emitted particles in the evening).

The available literature^43,53,83,85,86^ suggests that toxicologically active nanoparticles at urban sites may originate from both road transportation emissions and photochemically induced particle formation (i.e., photochemical oxidation of vehicular exhaust products yielding precursors for nucleation). Interestingly, not all the organic species of diesel exhaust particles have been found to be able to trigger inflammatory responses (comprised *CXCL-8* and *HMOX-1* upregulation) in exposed cells^87^. Studies targeting fresh combustion particles have shown that high *HMOX-1* values were associated with small soot particles due to their higher reactivity caused by their highly disordered internal structure^43^. With respect to photonucleation, high oxidation potential in the aerosol can be associated with short-lived free radicals, the occurrence of which has been demonstrated for the nanoparticles produced by the oxidation as furfural^83^. Noticeably, furan compounds can be formed by gas-phase reactions of hydrocarbons from gasoline and diesel emissions^88^.

Therefore, our findings suggest that it would be of great interest, in the future, to assess toxicity and detailed features of urban nanoparticles associated with a higher total DTT activity, and e.g. containing specific metals, such as Cu, and certain classes of reactive organic molecules, in the ambient air, at low PM$_1$ mass concentrations^16,22,23,72,75,80,89^.

### On the differential expression of the *HMOX-1* and *CXCL-8* genes

According to the specificity of the two genes analyzed, it should be kept in mind that *HMOX-1* is a gene often activated under a wide range of stressful conditions^90^ and that the transcription factor, Nuclear factor-erythroid 2-related factor 2 (Nrf2), regulates the expression of proteins functionally related to detoxification^91^ such as those in the antioxidant responsive elements, is responsible for the induction of *HMOX-1* transcription^92^. This activation is usually rapid and increased *HMOX-1* expression have been reported with a very short delay after (tens of minutes to few hours) cell treatments^93,94^. Moreover, *HMOX-1* has been recognized to be involved in controlling and eventually resolving inflammation^95^. On the contrary, *CXCL-8* gene expression depends on a sequence of biochemical events, usually starting from the activation of membrane receptors (toll-like receptors TLRs), that trigger the translocation of transcription factors into the nucleus and start gene transcription^96^, although TLRs independent stressors may induce upregulation of *CXCL-8*^97^.

Significantly for our experiments, the two genes taken together ideally represent two interconnected and, partially, consecutive steps of response of the lung epithelium to stress stimuli^58^, although specific insults may activate one or the other gene transcription independently. In fact, *HMOX-1* may precede and regulate inflammatory genes expression^98^ under stress conditions, although early *CXCL-8* upregulation is reported for crystalline silica particles^99^ in the absence of a related *HMOX-1* increased expression. In fact, the transcription of *CXCL-8* has been reported as a marker of inflammation and inflammatory diseases in the lungs^100^.

The differential expression of the two genes here reported is therefore related to the timing of expression of the two genes, considering also a strict interplay between different cellular response pathways, or to the response of the cells to specific stressors that activate response pathways leading to one or the other gene expression.

Finally, the expression of the *HMOX*-1 and *CXCL*-8 genes may be of relevance in explaining some of the lung diseases associated to PM exposure. The Adverse Outcome Pathway (AOP) approach in fact identifies the activation of oxidative and inflammatory responses in relation to lung diseases, such as lung carcinogenicity after nanoparticles exposure^101^ or COPD after cigarette smoke^102^ or lung fibrosis^103^. Our results, according to the reported AOPs link PM exposure also at really low concentrations to activation of key events that may lead to an adverse outcome in the lungs. Although further experiments are needed, the results here reported allow to define significant toxicological hazard of PM at concentrations usually considered as of not hazardous. Our results therefore suggest that new possible air quality guidelines should take into consideration new metrics and thresholds that are far below the ones in force so far

### The BC-to-OA ratio as an indicator of fresh combustion aerosols in the atmosphere

Even though the BC-to-OA presents lower predictive capacity than the “total DTT” and UFPs for *HMOX-1*, and even less for *CXCL-8*, it shows a certain correlation to the total DTT, the insoluble Cu, and N$_{8-20}$ (Fig. 1). Notably, the BC-to-OA can be derived by measurements that are more suitable for long-term and highly time- and spatial-resolved observations as compared to the OP and UFPs. Hence, it can be useful for monitoring purposes.

The BC-to-OA ratio was introduced as a proxy of combustion conditions^69^, with high BC-to-OA ratios indicating that combustion conditions are conducive to BC formation. The values of the BC-to-OA ratios observed in the current study (<0.5) refer to urban air mixtures, i.e. with both fossil fuel and biomass-burning aerosols. Interestingly, these do not necessarily translate into high BC (and/or EC) mass concentrations (Fig. S12 in SI Appendix shows that BC mass concentration being the same, the BC-to-OA ratio increases with decreasing d*p*).

Based on our findings and on previous studies^49,69–71,104^, here we interpret higher BC-to-OA ratios in the urban atmosphere as a rough indication of the availability in the atmosphere of combustion aerosols that have not had a long time to react and age in the atmosphere (less than one or a very few hours) and hence may be rich in redox-active components and particle-bound ROS in nanoparticles. Therefore, we suggest that fresh urban aerosols (as per Fig.4) may be initially identified by higher BC-to-OA ratios in the urban background. A number of factors, however, need to be clarified to understand a possible link between the BC-to-OA ratio and the OP and UFPs, as observed in Fig. 1. Key aspects include the enrichment of reactive organic compounds (like PAHs and their oxidation products) and of transition metals (our findings point particularly at the insoluble copper and its possible role for ROS formation), as well as the viscosity and physical state of these nanoparticles in the atmosphere^72,73,79–82,89,104^.

### The condensation sink in the atmosphere may influence ROS

Data show that an urban atmosphere with a low CS$_{200-900}$ can enhance the pro-inflammatory and anti-oxidant potency of the fine atmospheric aerosol. The ability of CS$_{200-900}$ to modulate gene expression patterns may be exemplary observed on 26, 27, 28 and 29 January in Fig. 3. We explain in Fig. 4 the influence of the CS$_{200-900}$ with different effects.

The availability of larger particles (high d$_p$ and high CS$_{200-900}$, Eq. 1) would suppress the traffic-related nucleation events, and the growth of the newly formed (fresh) particles. Pre-existing (larger) accumulation mode particles, if available, would favor the scavenging of available reactive gas phase compounds in virtue of their large surface area. This would suppress both their gas-to-particle conversion and/or their condensation upon smaller (more efficiently deposited in the lung) particles. Indeed, in a number of previous studies, low condensation sink has been found to favor particle formation^40,41^. A low CS$_{200-900}$ in the atmosphere can therefore modulate the interplay between specific toxic molecules and traffic-related nucleation mode particles, which are more efficiently lung deposited.

We believe that the role of the CS$_{200-900}$ in modulating inflammation and oxidative responses here may be linked also to the fact that high concentrations of preexisting particles can act as a condensation sink for the ROS molecules. We may interpret the CS$_{200-900}$ as an indicator of how rapidly ROS molecules are scavenged by preexisting larger aerosol particles, which have a lower lung deposition efficiency. This is supported by the inverse relation between CS$_{200-900}$ and the particle-bound ROS (Fig.3). Given the relationship between the CS and d$_p$ (Eq. 1), we interpret a low CS$_{200-900}$ value as an increased possibility for a ROS molecule to be bound on nanoparticles. Further mechanisms linked to the chemistry of nanoparticles may be relevant.

### To what degree PM$_{1}$ mass and OP relate to the observed oxidative stress and inflammation

**Figure 5:**
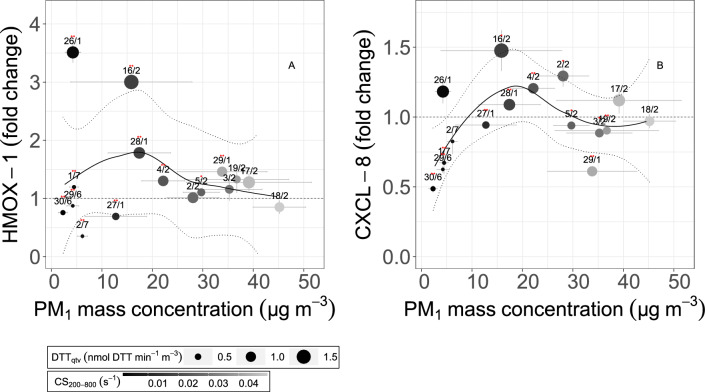
Concentration-response curves for PM$_1$ mass concentration versus pro-oxidative and inflammation biomarkers. Gene expression patterns for *HMOX*-1 (indicative of oxidative stress, panel **A**) and *CXCL*-8 (indicative of inflammation, panel **B**) are indicated against PM1 mass concentration (x-axis). Data points are colored by the condensation sink, and sized by the aerosol Oxidative Potential (total DTT activity in PM_1_). The fitted regression lines are indicated with a 95% confidence interval (dashed ribbons). Data points are indicated with measurement date (dd/m), standard deviation error bars, and significance level of the biomarkers (** indicate *p*-value < 0.01, * indicates *p*-value < 0.05).

Regression lines in Fig. 1 provide evidence of non-linear effects between PM$_1$ mass concentration and both *HMOX-1* and *CXCL-8* gene expression patterns. In Fig. 5 we show as these relations are modulated by the values of CS$_{200-900}$ and OP. PM$_1$ mass being the same, both *HMOX-1* and *CXCL-8* f.c. increase with decreasing the CS$_{200-900}$ and OP (Fig. 5C, D).

The larger PM$_1$ mass concentrations (at least in our dataset) are mainly associated to a prevalence of the *urban aged aerosol* (Fig. 4). This is low in the BC-to-OA ratio (being aged), and high in the CS$_{200-900}$ (due to the larger particle diameter), an argument for the low gene expression values observed. The total OP values may be high in this case, as observed on 29/01, 18/02, and 19/02, albeit the toxicological markers observed are low. At PM$_1$ mass <10-20 $\upmu$g m$^3$, the urban aerosol is shortly aged in the atmosphere (higher BC-to-OA ratios) and dominated by smaller particles with a lower CS$_{200-900}$: PM$_1$ mass being the same, oxidative stress and inflammation gene expression values increase with increasing the total OP. Patterns among PM$_1$ and toxicological markers show steeper slopes and higher values at lower PM$_1$ mass concentrations, as well as a change of slope in the range of 10-20 $\upmu$g m$^3$. For PM$_1$ mass concentration < 20 $\upmu$g m$^3$, a positive correlation between PM$_1$ mass and *CXCL-8* (and less significantly with *HMOX-1*) can be observed, not at higher loading. Most interestingly, such correlation is modulated by the combination of CS$_{200-900}$ and OP (Figs. 5, and S12, SI).

Therefore, our analysis highlights that there is no single aerosol variable able to predict the overall complexity of the oxidative stress/inflammatory responses. However, we found that a combination of two aggregate indicators of aerosol physicochemical properties (the OP mainly for chemistry, the CS mainly for microphysics) may explain simultaneously most of the modulation of the two genes here observed taken together.

### On the low PM_1_ mass concentration and low doses

Data of the current study were taken through a recently developed methodology to limit artifacts in lung exposure to urban UFPs in the atmosphere^35–37^. Accordingly, we could cover the low range of PM$_1$ concentrations (down to less than 2 $\upmu$g m$^{-3}$) reflecting the near - background global PM$_{2.5}$ concentrations now addressed by the last WHO guidelines for outdoor PM$_{2.5}$^8^. This is the range where the understanding of the concentration-response relationship for PM$_{2.5}$ and mortality remains incomplete because most of the literature is based on high PM$_{2.5}$ concentrations^7,8^.

Our study suggests a model that may explain exposure to the PM$_{2.5}$ in the urban environment, especially at low PM$_{2.5}$ mass concentrations, and makes connections to recently found relationships in low-exposure cohorts, where similar findings are observed, but reasons are still to be found^1,3,7,9,12,77^

Consistent with the existing literature^1,3,5–8,11,13,43,76,77,105^, our findings indicate no basis to assume a PM$_1$ mass concentration threshold below which neither *HMOX-1* nor *CXCL-8* are low: conversely, there is a significant probability of toxicological markers to be expressed at the very low doses of PM1 exposures if these are accompanied by high concentrations of ROS-rich UFPs. This may be the case of Copper, for which our analysis suggests a relation between insoluble Cu mass concentration, nanoparticles and the antioxidant gene HMOX-1 and the proinflammatory gene CXCL-8, which is stronger than that observed for other metals (Figs. S3–S4 in the SI). We may interpret the finding in coherence with recent literature demonstrating biological effects, in vitro and in vivo, stronger for Cu and Cu-doped nanoparticles (as compared to Fe), on both the antioxidant and the inflammatory response pathways, at low doses of exposure - as low as those observed here (hundreds of nanograms per square cm)^74,75^.

## Conclusions

Our findings suggest that urban nanoparticles enriched in the redox-active compounds traced by the “total DTT” assay (such as insoluble copper) may have high pro-inflammatory and oxidative responses at low PM$_1$ concentrations (< 5 $\upmu$g m$^{-3}$). This could be associated with the enrichment in traffic-related nanoparticles of reactive compounds otherwise scavenged by larger particles (associated to higher PM$_1$). In fact, these larger particles have a lower deposition efficiency in the lungs as compared to nanoparticles. We found no basis to assume a safe exposure limit for PM$_1$ below which pro-inflammatory and oxidative responses are low. These findings support a new conceptual model contributing to explaining the mechanisms governing human exposure to ambient fine aerosol, especially at low doses. Possible explanations for PM$_1$ concentration-response curves are obtained by involving the interplay between OP and CS. Once larger particles are low (lower PM$_1$, and CS), the condensed redox-active organic compounds may become enriched in nanoparticles. When these nanoparticles are abundant and already contain reactive compounds like trace metals (Copper), the strongest toxicological response can be observed. A similar condition might typically occur in urban areas during the traffic rush hour of a winter weekday, immediately after bad weather conditions.

Among the implications of this work, there are recommendations for new air quality guidelines. Global efforts should go toward the refinement of actual air quality standards also at relatively low PM$_{2.5}$ mass concentrations, a space where the urban nanoparticles may have a peculiar role. This study joins other previous studies that call for measuring aerosol physicochemical properties rather than just PM$_{2.5}$ mass concentration^7^ , since these measurements may be more closely related to the actual biological outcomes. Air quality metrics should more closely proxy the specific aerosol features connected to relevant toxicological markers endpoints, rather than the bulk aerosol (i.e., total mass, total number concentration). We highlight the importance to consider the oxidative potential (“total DTT” assay) in PM$_1$ to trace aerosol toxic effects, suitable for monitoring. We add to this the suggestion to consider the Condensation Sink as a function of particle size, as a new proxy for the ability of large particles to scavenge reactive compounds otherwise prompted to condense on ultrafine and nanoparticles, which efficiently deposit on alveolar tissues. Our results, finally, confirm the potential importance of having air quality metrics considering the air mixture properties, and specifically how redox-active compounds distribute in, and interact with, fine and ultrafine particles.

### Supplementary Information

Supplementary Information.

## Data Availability

The datasets analysed during the current study are available in the RHAPS repository (https://campagne.isac.cnr.it/RHAPS/ [username: rhaps, password: qui3Ahz!]), and available from the corresponding author upon reasonable request.
