# Supplementary material for: Exposure to urban nanoparticles at low PM1 concentrations as a source of oxidative stress and inflammation
Source: Sci Rep. 2023 Oct 30;13:18616. doi: 10.1038/s41598-023-45230-z (PMC10616204; doi:10.1038/s41598-023-45230-z)
Supplement: Supplementary file 1 — Supplementary Information. [file 41598_2023_45230_MOESM1_ESM.pdf]

Supporting Information for

Exposure to urban nanoparticles at low PM1 concentrations as a source of oxidative stress and inflammation

F. Costabile, M. Gualtieri, M. Rinaldi, S. Canepari, R. Vecchi, L. Massimi, G. Di Iulio, M. Paglione, E. Corsini, L. Di Liberto, C. Facchini, and S.Decesari

Corresponding Author name.
E-mail: f.costabile@isac.cnr.it

This PDF file includes:

Figs. S1 to S12
Tables S1 to S3
SI References

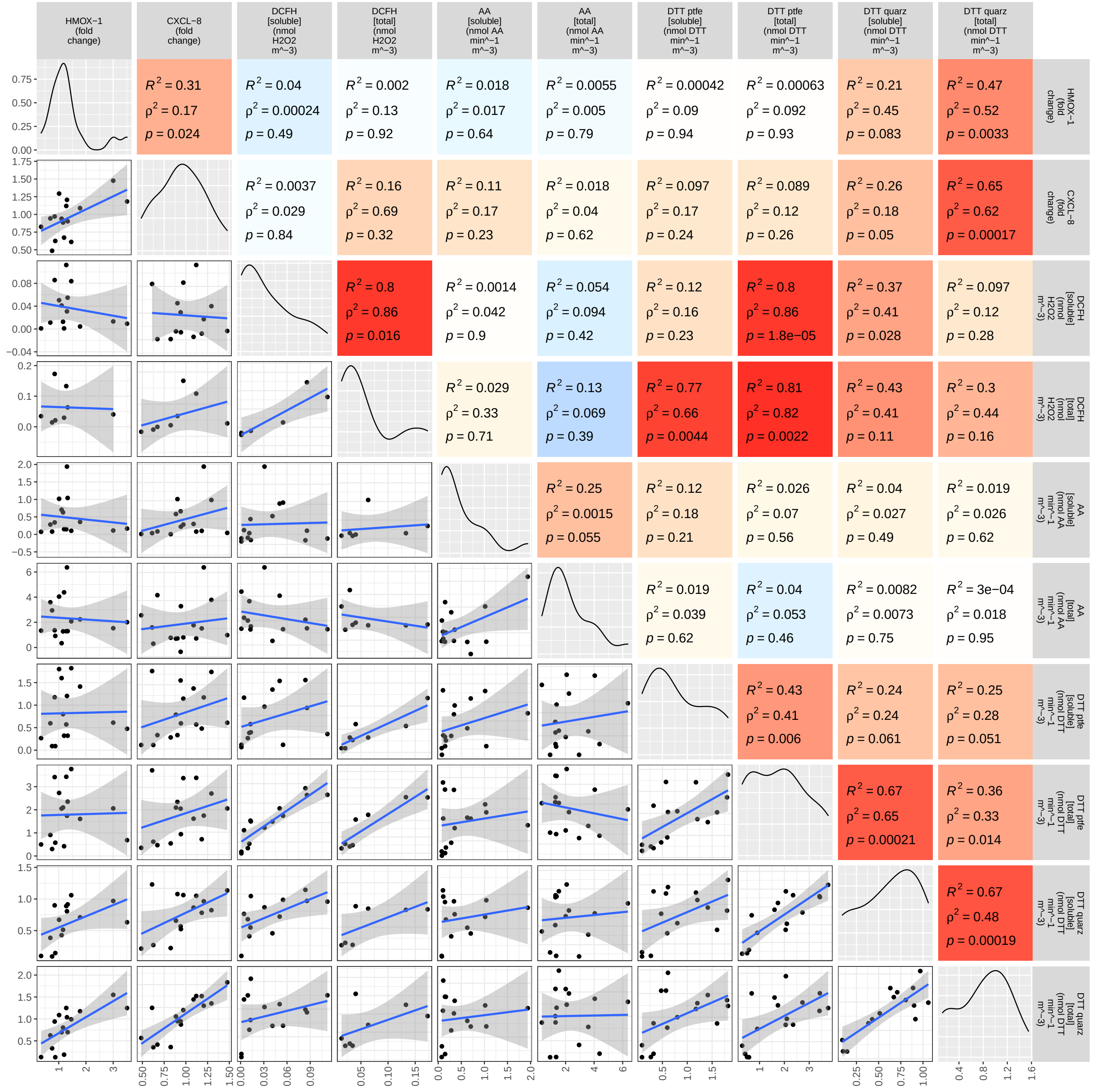

Fig. S1. Statistics for comparison between the oxidative potential (OP) in PM1 and the toxicological markers for oxidative stress and inflammation. Paired scatterplot matrix shows 24-hour data for variables related to the OP by different assays, DTT (PTFE and quartz filter), AA, DCFH, both water-soluble and total, and HMOX1 and CXCL8 gene expression. Pearson's and Spearman's correlation coefficients are indicated in the box, with relevant statistical significance (p-value). Color gradient is proportional to Pearson's correlation coefficient. Number of points (n) to calculate correlations is 16. Created in R-Studio version 2022.12.0 using ggpairs function from the GGally package version 2.1.2.

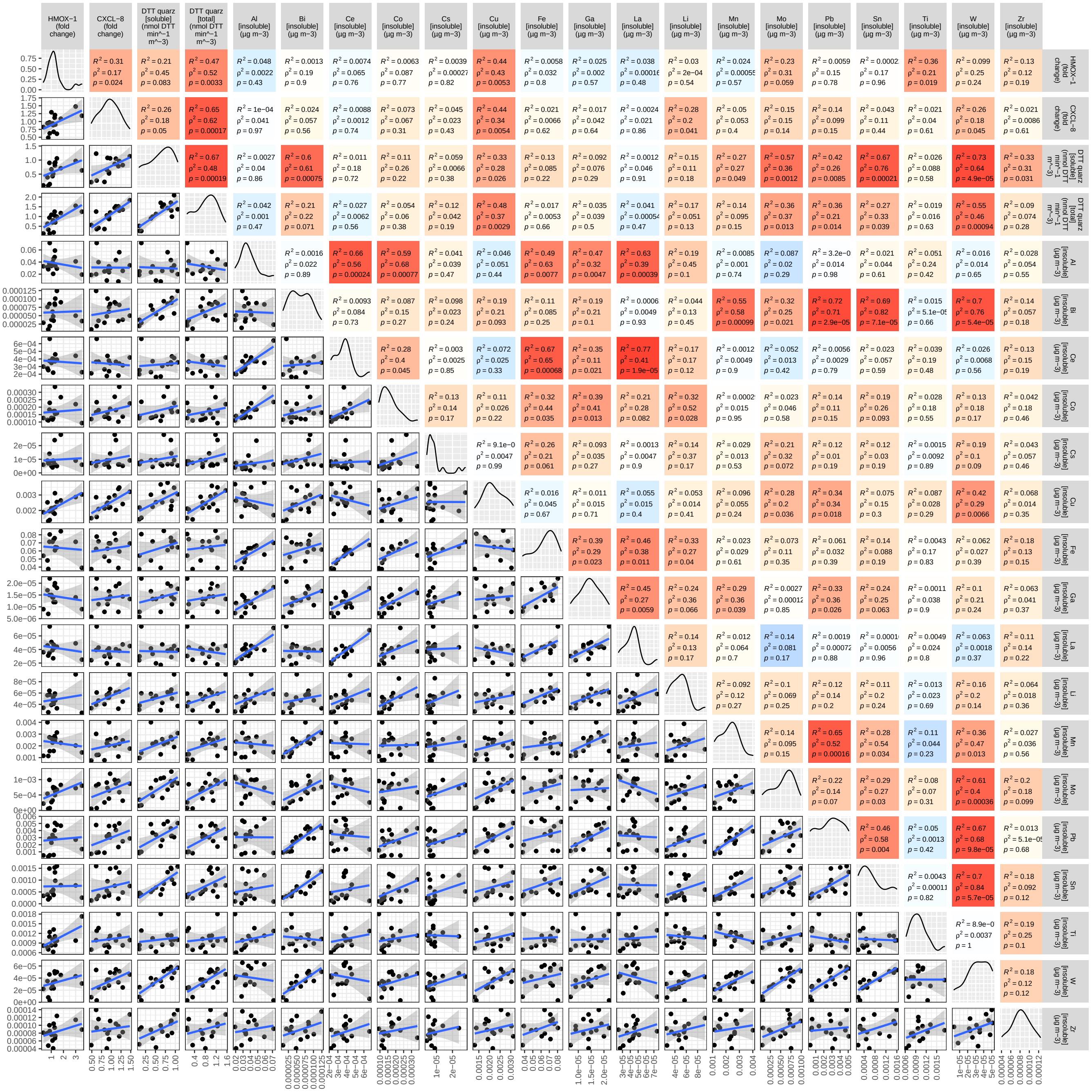

Fig. S2. Statistics for comparison between insoluble metals in PM1 and related pro-inflammatory and oxidative toxicological markers during the 16 days of observations. Paired scatterplot matrix shows 24-hour data for variables related to the insoluble metal mass concentration and toxicological markers for oxidative stress (fold change in gene expression for HMOX-1) and inflammation (CXCL-8). Pearson's and Spearman's correlation coefficients are indicated in the box, with relevant statistical significance (p-value). Color gradient is proportional to Pearson's correlation coefficient . Number of points (n) to calculate correlations is 16. Created in R-Studio version 2022.12.0 using ggpairs function from the GGally package version 2.1.2.

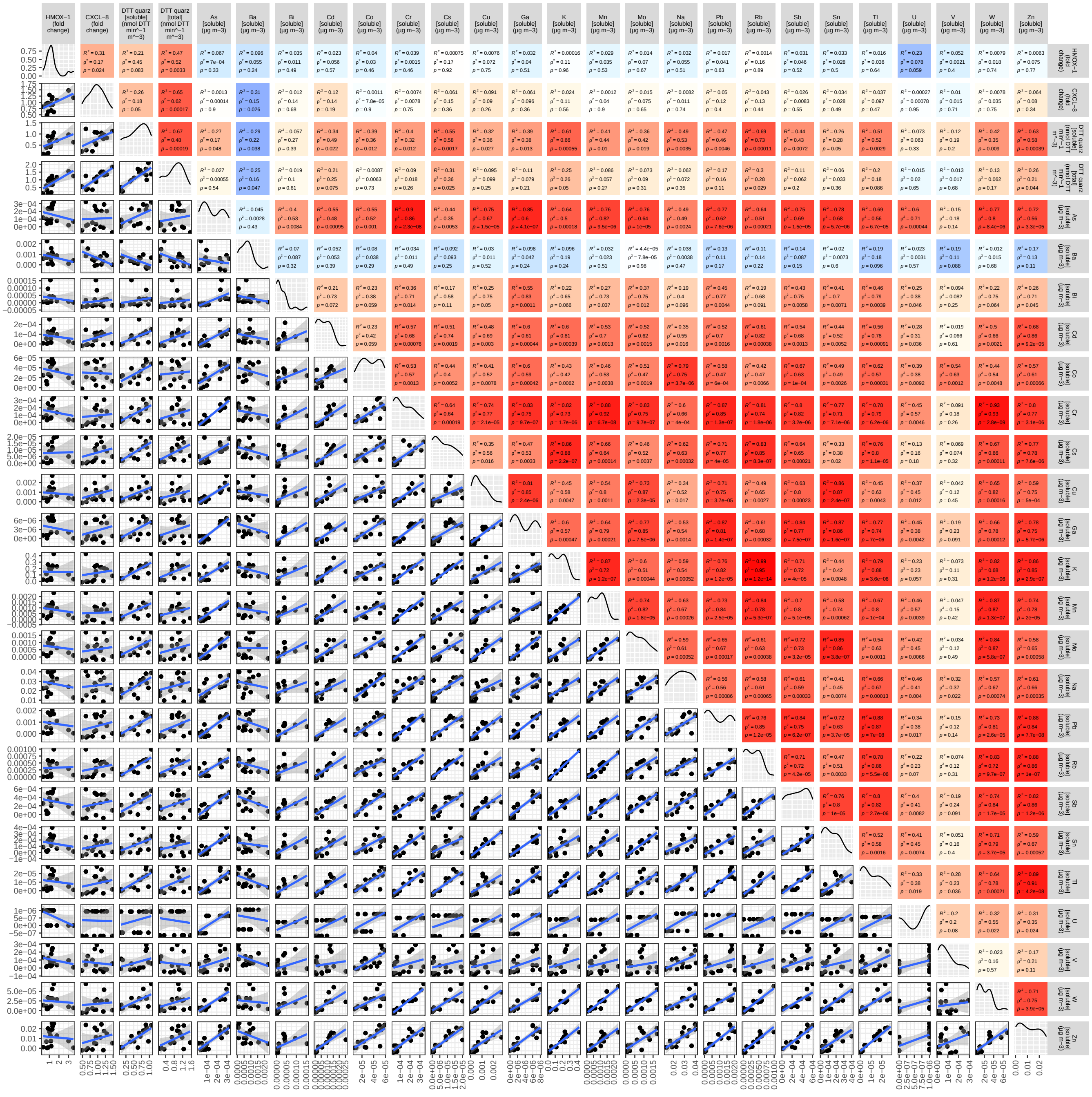

Fig. S3. Statistics for comparison between soluble metals in PM1 and related pro-inflammatory and oxidative toxicological markers during the 16 days of observations. Paired scatterplot matrix shows 24-hour data for variables related to the soluble metal mass concentration and toxicological markers for oxidative stress (fold change in gene expression for HMOX-1) and inflammation (CXCL-8). Pearson's and Spearman's correlation coefficients are indicated in the box, with relevant statistical significance (p-value). Color gradient is proportional to Pearson's correlation coefficient . Number of points (n) to calculate correlations is 16. Created in R-Studio version 2022.12.0 using ggpairs function from the GGally package version 2.1.2.

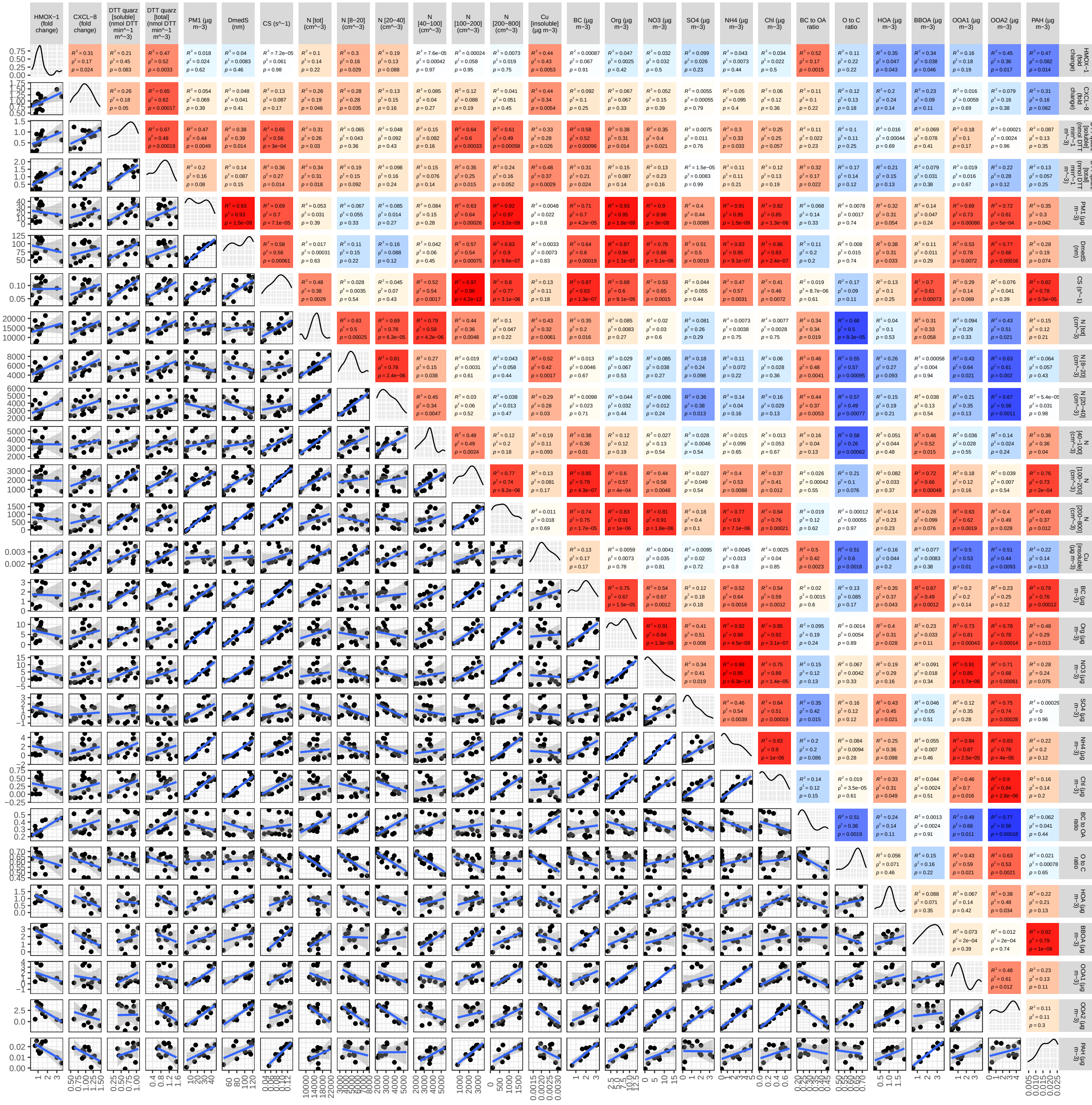

Fig. S4. Statistics for comparison between several aerosol variables in PM1 and related pro-inflammatory and oxidative toxicological markers during the 16 days of observations. Paired scatterplot matrix shows 24-hour data for variables related to DTT (water soluble and total), PM1 mass concentration (total, BC, organics, nitrates, sulfates, ammonium, chloride, Hydrocarbon-like Organic aerosol, HOA, biomass burning OA, oxidised OOA1 and OOA2), particle number concentration in selected size ranges and toxicological markers for oxidative stress (fold change in gene expression for HMOX-1) and inflammation (CXCL-8). Pearson's and Spearman's correlation coefficients are indicated in the box, with relevant statistical significance (p-value). Color gradient is proportional to Pearson's correlation coefficient . Number of points (n) to calculate correlations is 16. Created in R-Studio version 2022.12.0 using ggpairs function from the GGally package version 2.1.2.

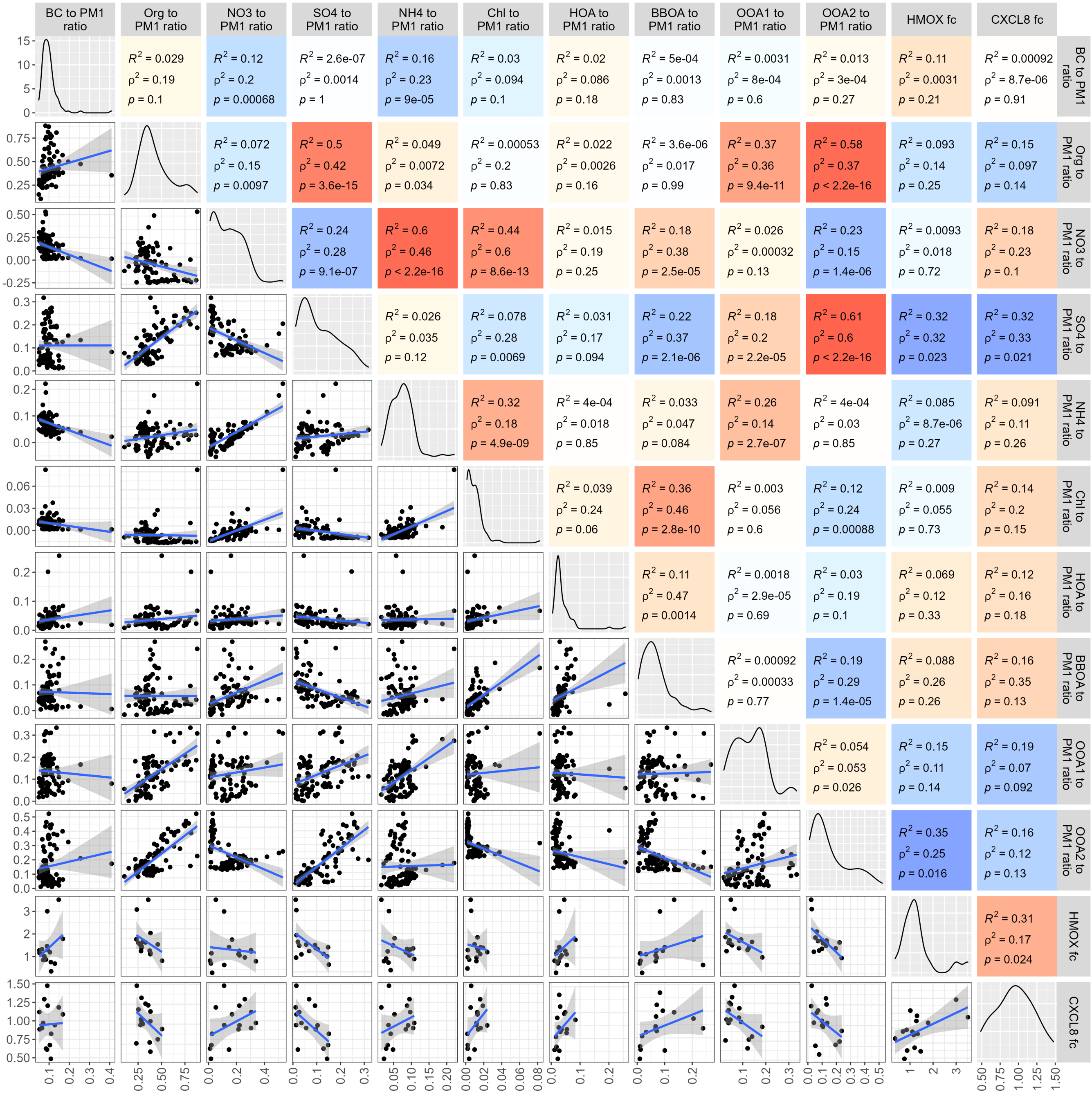

Fig. S5. Statistics for comparison between speciated PM1 mass fractions and related pro-inflammatory and oxidative toxicological markers. Paired scatterplot matrix shows 24-hour data for variables related to PM1 mass fractions (organics, nitrates, sulfates, ammonium, chloride, Hydrocarbon-like Organic aerosol, HOA, biomass burning OA, oxidised OOA1 and OOA2) and biological endpoints for oxidative stress (fold change in gene expression for HMOX-1) and inflammation (CXCL-8). Pearson's and Spearman's correlation coefficients are indicated in the box, with relevant statistical significance (p-value). Color gradient is proportional to Pearson's correlation coefficient . Number of points (n) to calculate correlations is 16. Created in R-Studio version 2022.12.0 using ggpairs function from the GGally package version 2.1.2.

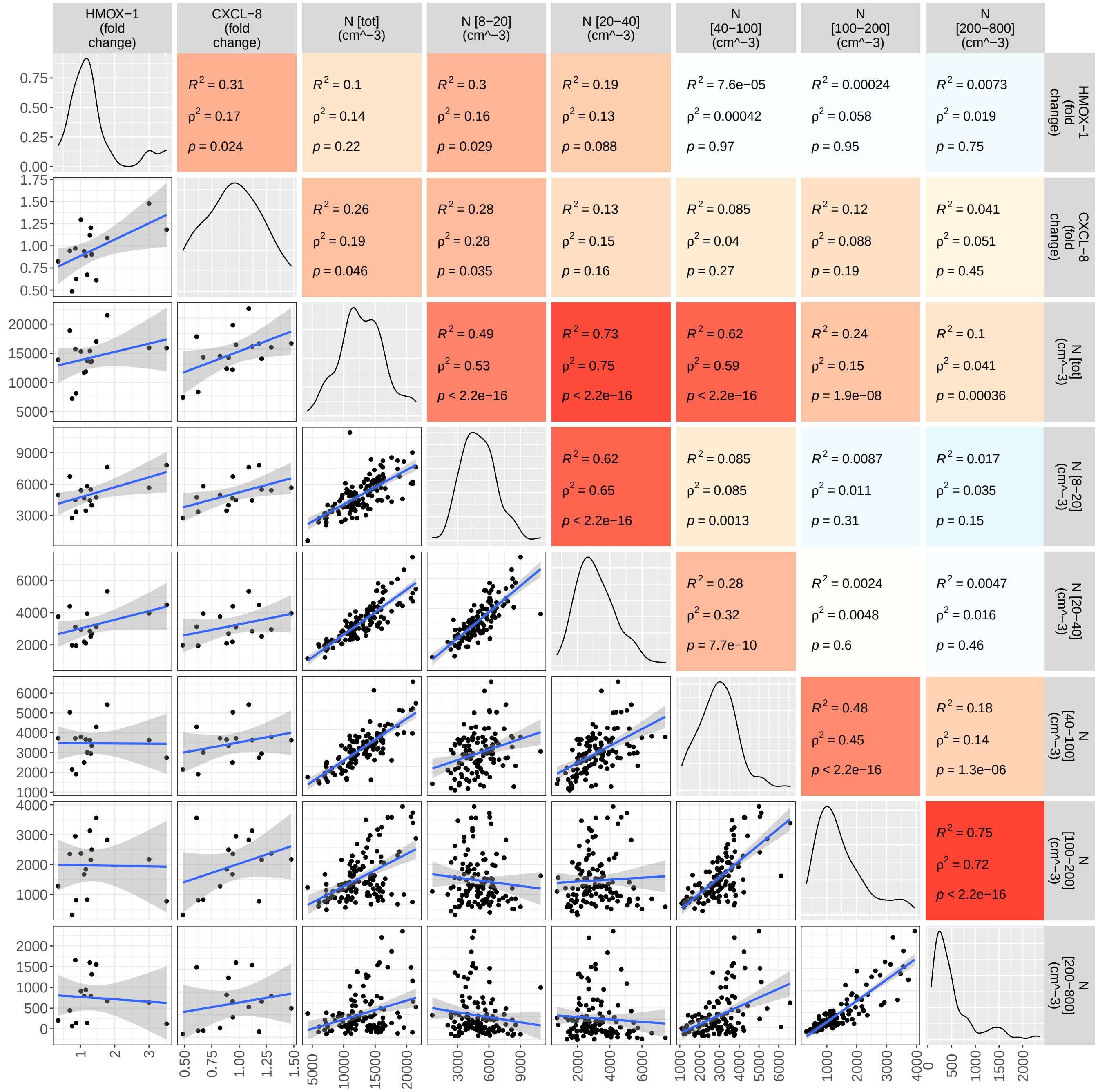

Fig. S6. Statistics for comparison between number concentrations (N) in selected particle size ranges and related pro-inflammatory and oxidative toxicological markers. Paired scatterplot matrix shows 24-hour data for nucleation mode (N_{8-20}), Aitken mode (N_{20-40}), soot mode (N_{40-100}), condensation mode ($N_{100-200}$) and accumulation mode ($N_{200-900}$) and toxicological markers for oxidative stress (fold change in gene expression for HMOX-1) and inflammation (CXCL-8). Pearson's and Spearman's correlation coefficients are indicated in the box, with relevant statistical significance (p-value). Color gradient is proportional to Pearson's correlation coefficient . Number of points (n) to calculate correlations is 16. Created in R-Studio version 2022.12.0 using ggpairs function from the GGally package version 2.1.2.

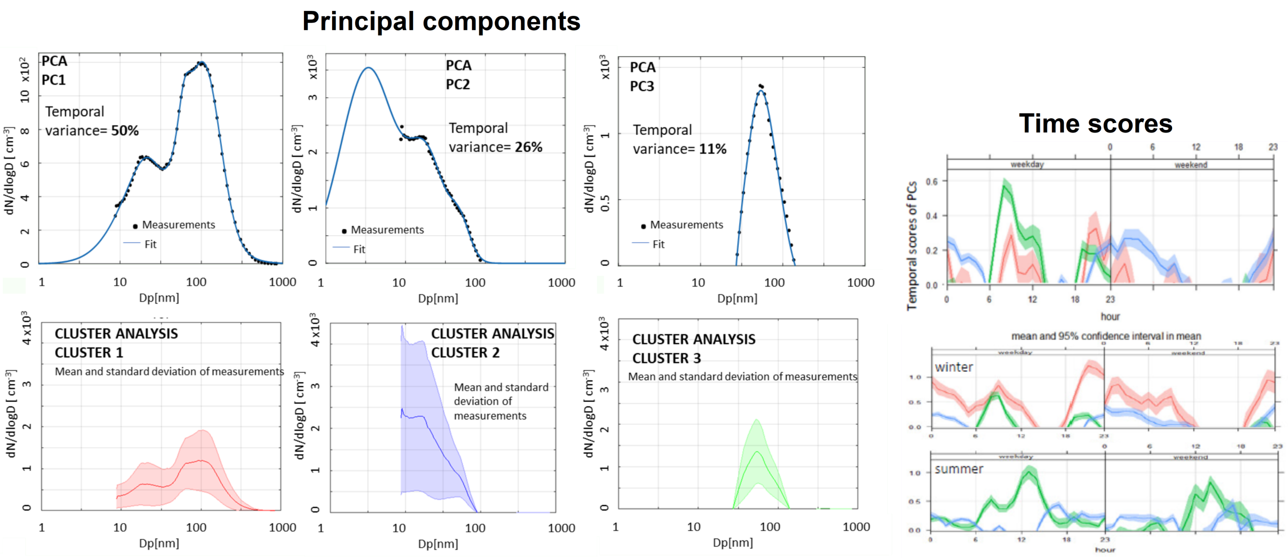

Log-normal distribution fitting

PC	mode 1			mode 2			mode 3			r^2 fit
	µ [nm]	σ [nm]	N [# cm^{-3}]	µ [nm]	σ [nm]	N [# cm^{-3}]	µ [nm]	σ [nm]	N [# cm^{-3}]	
PC1	36.3±2.8	2.65±0. 10	831±1.3	61.5±1.3	1.16±0.03	195±40	115.6±2.5	1.69±0.02	1212±10	0.99
PC2	18.5±2	1.69±4	1825±2e+04	48.4±18.4	1.3±0.50	606±1e+03	n.a.	n.a.	n.a.	0.99
PC3	n.a.	n.a.	n.a.	46.2±1.6	1.81±0.07	2404±215.5	n.a.	n.a.	n.a.	0.99

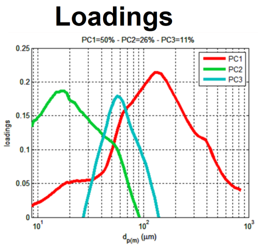

Fig. S7. Statistical analysis of the Particle Number Size Distributions. Panels show principal components extracted by Principal Component and Clustering Analysis, and time scores daily/weekly cycles, loadings, and variables of the log-normal distribution fitting for these components. Data cover the entire period of the RHAPS experiment, with a 10-minute temporal resolution.

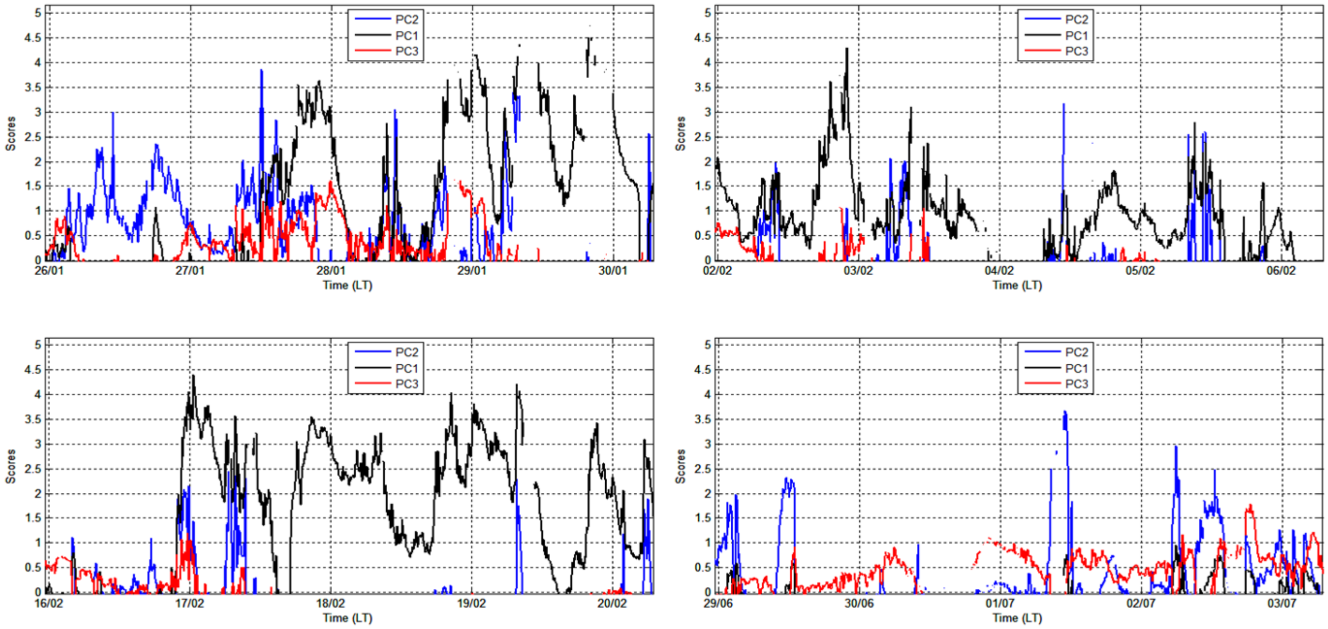

Fig. S8. Temporal variability of the principal components of the Particle Number Size Distributions. Panels show time-series of the time scores for the three principal components presented in Fig.S1.

Multiple regression output	Coefficient	SE	P value
**HMOX-1 = -28.0 CSacc + 1.7 OP_t^{DTT} + 0.5**			
Adjusted R^2 0.69, Multiple R^2 0.71, Regression ANOVA: p-value<0.001			
Intercept	0.5459	0.2636	0.05883 .
CSacc	-28.2494	-3.310	0.00564 **
DTTt	1.7775	5.627	8.24e-05 ***
**CXCL-8 = -4 CSacc +0.6 OP_t^{DTT} + 0.6**			
Adjusted R^2 0.64, Multiple R^2 0.69, Regression ANOVA: p-value<0.001			
Intercept	0.5818407	0.09052	2.24e-05 ***
CSacc	-3.86401	2.93113	0.0210170 .
DTTt	0.56535	0.10848	0.000168 ***

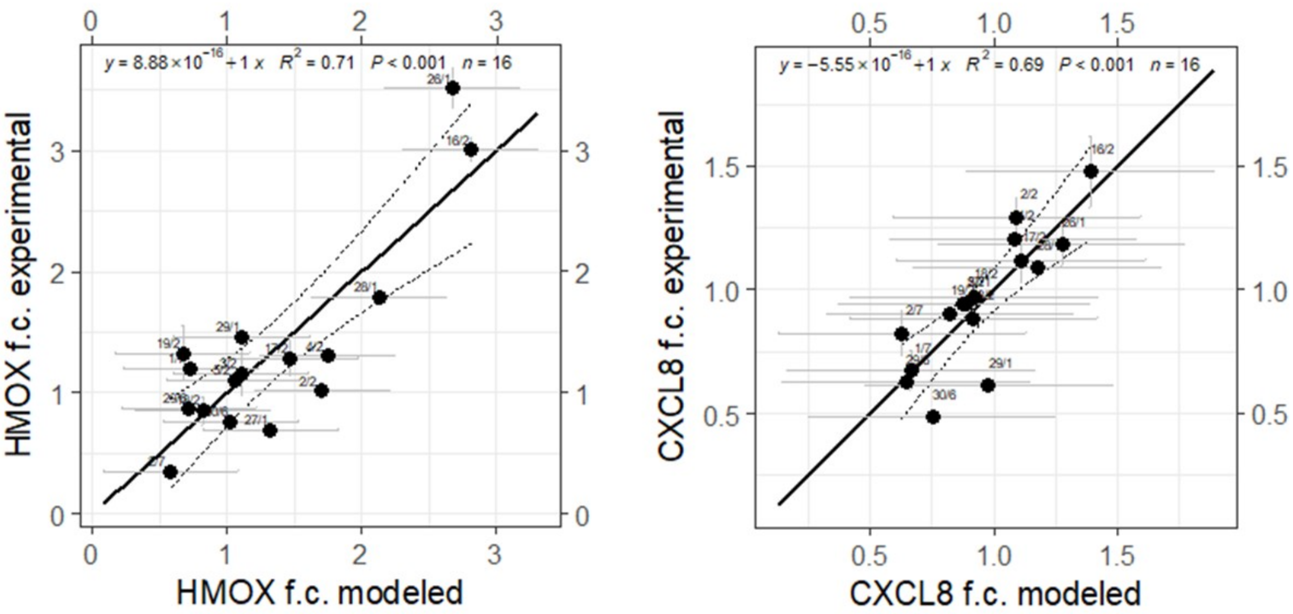

Fig. S9. Multiple regression linear models to identify major contributions from the aerosol variables investigated on the HMOX1 and CXCL8 gene expression. The model shows the total oxidative potential (OP) by the DTT assays (nmol DTT /min /m3)) and the Condensation Sink (CS) of the accumulation mode in unit per second as predictive variables simultaneously for both HMOX1 and CXCL8. Created in R-Studio version 2022.12.0 using package lm to fit a linear model.

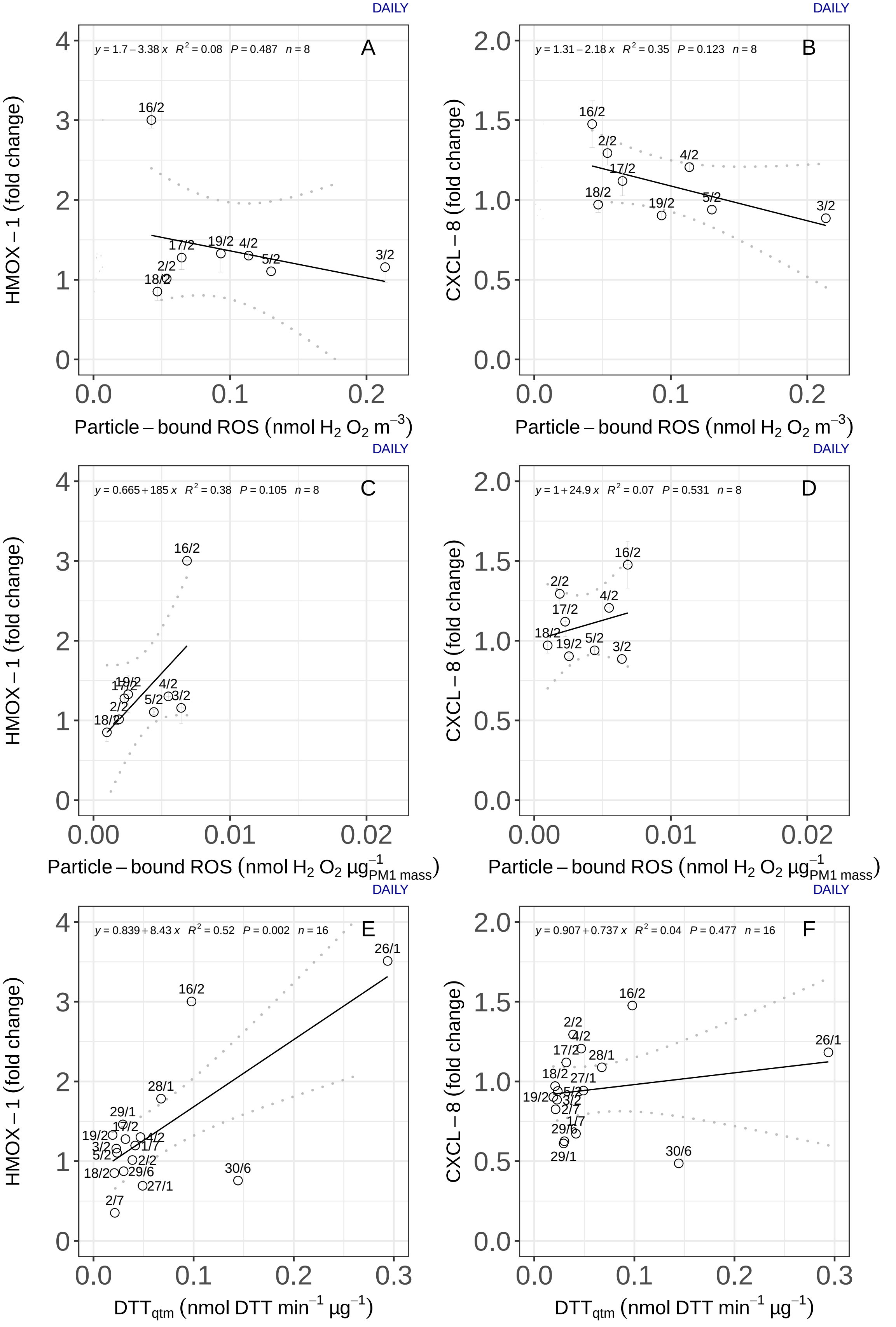

Fig. S10. Relation between HMOX1 and CXCL8 gene expression and the particle-bound ROS concentration (panels A and B, while panels C and D show relation with the mass-normalized values) and DTT on a per volume basis.

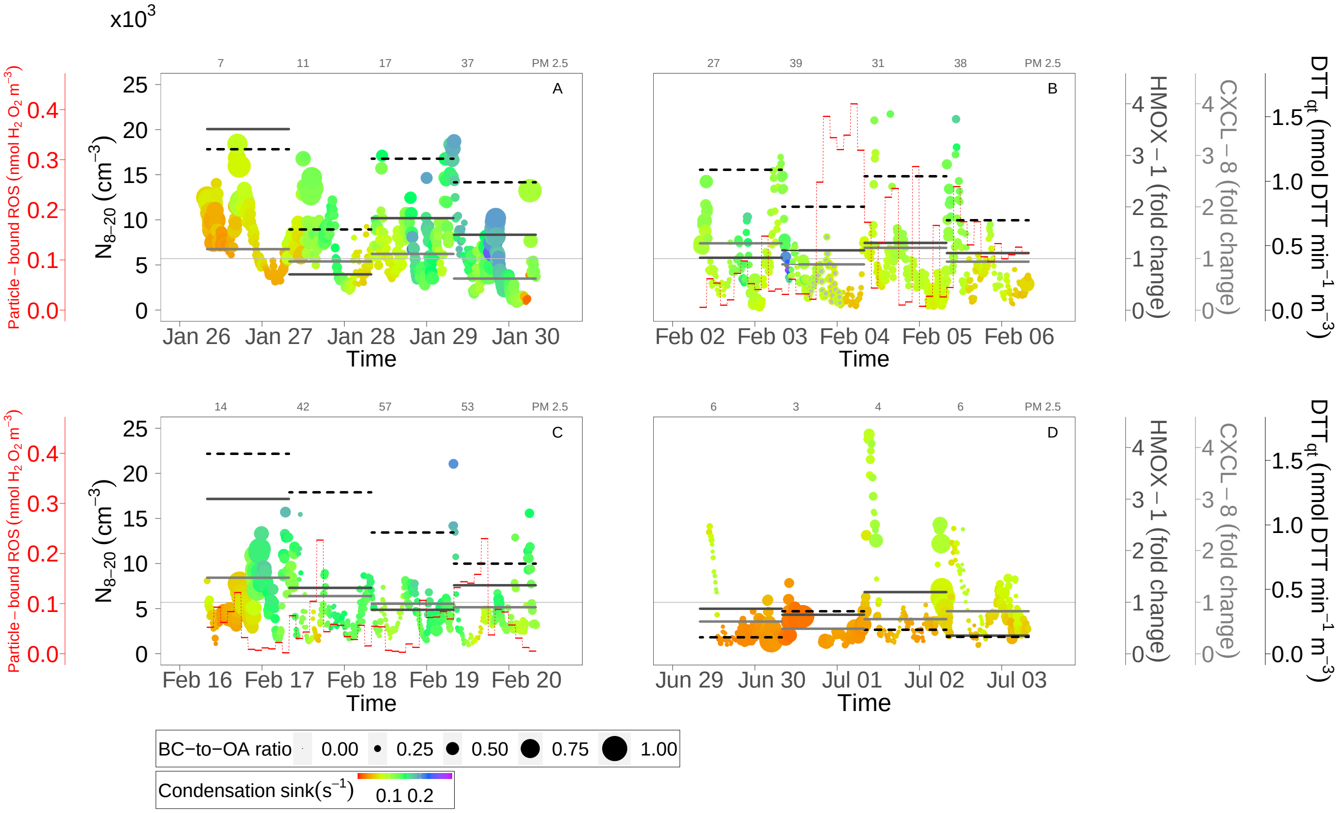

Fig. S11. Same as Figure 3 in the main text, except for particle-bound ROS (DCFH) expressed as volume-normalized values.

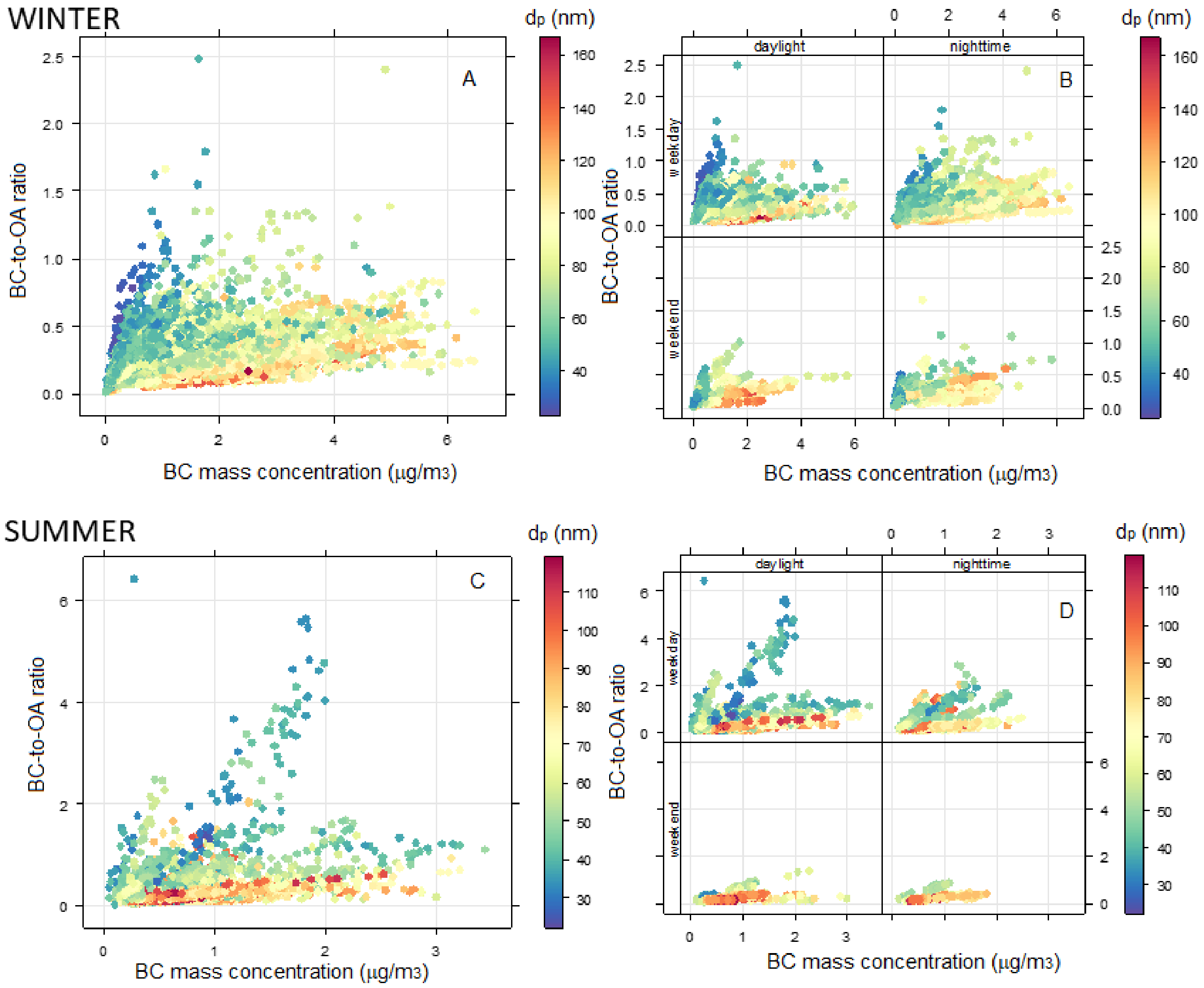

Fig. S12. Relationship between the BC mass concentration and the BC-to-OA ratio. Scatter plots show the BC mass concentration vs. the BC-to-OA ratio in winter and summer, separately for the weekday and weekends, and daylight and nighttime. Markers are colored by the median diameter of the particle number size distribution. Data cover the entire period of the RHAPS experiment, with a 10-minute temporal resolution.

Table S1. Daily values of PM1 and BC mass concentration. 24-hour averaged PM1 and BC mass concentration measured during the exposure days.

DAY 2021	PM1 $(\mu g \cdot m^{-3})$	BC $(\mu g \cdot m^{-3})$
Jan 26	4±1	0.6±0.3
Jan 27	13±6	1.6±0.8
Jan 28	17±6	2.9±1.5
Jan 29	34±9	3.0±1.1
Feb 02	28±5	2.1±0.6
Feb 03	35±6	1.9±0.6
Feb 04	22±4	2.1±0.3
Feb 05	30±4	1.9±0.6
Feb 16	16±12	1.4±0.9
Feb 17	39±12	2.0±0.6
Feb 18	45±5	3.2±1.4
Feb 19	37±10	2.4±0.9
Jun 29	4±1	0.3±0.1
Jun 30	2±1	0.2±0.1
Jul 01	4±1	0.5±0.4
Jul 02	6±1	0.7±0.3

Table S2. Correlation table between gases and genes. Pearson correlation coefficient is shown, while the Spearman correlation coefficient is in brackets. No p-value lower than 0.05 was found. The number of points is 16, including 12 winter and 4 summer points.

Genes	NO_2	Ozone
HMOX-1	-0.05 (-0-03)	0.08 (-0.12)
	p>0.05	p>0.05
CXCL-8	0.17 (0.3)	0.04 (0.13)
	p>0.05	p>0.05

Table S3. Reference to the literature

ID	Aerosol with high endpoint	Biol.Endpoint	Methods	Ref
I	fresh traffic-related UFPs	HMOX-1 gene	atmosphere, ALI	(1)
II	SOA-coated soot	MDA gene	laboratory, ALI	(2)
III	Diesel exhaust	HMOX-1, NQO-1, CXCL8	diesel generator, ALI	(3)
IV	UFPs, low mass concentration	HMOX gene	engine (only non-volatile), ALI	(4)
V	smaller size, organic coatins on BC, ageing	COMET	laboratory, ALI	(5)
VI	traffic quasi-UFPs	HMOX gene	atmosphere, filters	(6)
VII	anthropogenic sources of PM	HMOX,NQO1 gene	filters, ALI	(7)
VIII	small insoluble particles	HMOX gene	engine, filters	(8)
IX	Diesel engine exhaust particles	ROS	laboratory, filters	(9)
X	short-lived unsuturated carbonyls in fresh nSOA	proteome	laboratory, filters	(10)
XI	photoactive organic particles containing iron	ROS, CCR	laboratory, model	(11)

1. Limitations for OP measurements

Each of the three OP methods used in the present study (DTT, AA, DCFH) has been deemed sensitive to PM from different emission sources and characterized by very different physico-chemical properties (12–14). DTT is considered a chemical surrogate for cellular reductants (such as NADH or NADPH) that reduce O2 to superoxide anion (O2 ·) and induce oxidative stress. The AA used in the test is a physiological antioxidant that prevents the oxidation of lipids and proteins. DDT and AA assays involve the controlled incubation of the antioxidant (DTT or AA) in aqueous extracts of PM under controlled conditions and measuring their depletion over time (15–17). The rate of antioxidant loss represents the ability of redox-active species in the aerosol to transfer electrons from DTT or AA to oxygen. In contrast, the DCFH assay was developed in the past for the in-vitro determination of ROS in biological cells, but has been adapted and applied as an acellular method in recent years (15–17). In this assay, non-fluorescent DCFH is oxidized to fluorescent dichlorofluorescein (DCF) by ROS in the presence of horseradish peroxidase (HRP). The fluorescence of the formed DCF can be easily measured at excitation and emission wavelengths. Although these methods are often applied to predict the biological effects of PM, there is still a gap of knowledge on the relationship between the concentration and chemical composition of PM and the oxidative potential results obtained with these assays (12, 13, 16, 17). Each of these methods has been deemed sensitive to PM from different emission sources and characterized by very different physic-chemical properties (12–14). Therefore, none of the OP assays can be a-priori considered as representative of ROS generation pathways in biological organisms. In numerous studies in the literature and in previous studies that we carried out (12–15, 17), it appears that each of these assays is sensitive to particles with different size distribution and chemical composition. The AA assay seems to be particularly sensitive toward coarse particles (1.8-10 m) released from non-exhaust traffic (12, 13, 15). This source releases particles rich in some transition metals, such as Cu, Fe and Mn, and AA has been strongly positively correlated with the main elements tracing non-exhaust traffic emissions (13, 16). The DTT assay was found to be specifically sensitive toward the fine fraction of PM (0.18-1.8 m) released by both industrial and biomass burning sources, such as domestic biomass heating. Several studies have shown strong correlations of DTT with transition metals (such as Cu, Fe and Mn) and with robust biomass combustion tracers such as K and organic compounds like levoglucosan (12, 13, 16). In contrast, regarding the DCFH assay, positive correlations were found between ROS and concentrations of transition metals (including Fe) and organics (13, 16); overall, the DCFH assay appeared to be particularly sensitive to fine particles (0.18-1.8 m) released by combustive processes from industrial, biomass burning and vehicular traffic sources. As there are still doubts and uncertainties regarding the most representative assay to quantify PM oxidative potential, the synergistic application of different acellular methods on the same PM sample is often considered advantageous to provide an in-depth assessment of the particles' OP.

References

1. F Costabile, et al., First results of the "carbonaceous aerosol in rome and environs (care)" experiment: Beyond current standards for pm10. *Atmosphere* **8** (2017).
2. Offer, Effect of atmospheric ageing on soot particle toxicity in lung cell models at the air liquid interface: Differential toxicological impacts of biogenic and anthropogenic secondary organic aerosols (soas). *Env Heal. Perp* **130**, 2 (2022).
3. M Zarcone, et al., Cellular response of mucociliary differentiated primary bronchial epithelial cells to diesel exhaust. *Am J Physiol Lung Cell Mol Physiol* **311**, L111–123 (2016).
4. HR Jonsdottir, M Delaval, Z Leni, al, Non-volatile particle emissions from aircraft turbine engines at ground-idle induce oxidative stress in bronchial cells. *Commun Biol.* **2**, 90 (2019).
5. H Hakkarainen, et al., Black carbon toxicity dependence on particle coating: Measurements with a novel cell exposure method. *Sci. The Total. Environ.* **838**, 156543 (2022).
6. US Akhtar, N Rastogi, RD Mcwhinney, The combined effects of physicochemical properties of size-fractionated ambient particulate matter on in vitro toxicity in human a549 lung epithelial cells. *Hum. Nat.* **20**, 317–330 (2014).
7. Z Leni, et al., Oxidative stress-induced inflammation in susceptible airways by anthropogenic aerosol. *PLoS One* **18**, 15(11):e0233425 (2020).
8. J Yang, al, Physical, chemical, and toxicological characteristics of particulate emissions from current technology gasoline direct injection vehicles. *Sc. Tot. Environ.* **650**, 1182–1194 (2019).
9. M Park, HS Joo, a Lee, Differential toxicities of fine particulate matters from various sources. *Sci Rep* **8**, 17007 (2018).
10. J Han, al, Proteome-wide effects of naphthalene-derived secondary organic aerosol in beas-2b cells are caused by short-lived unsaturated carbonyls. *Proc. Natl. Acad. Sci.* **117**, 25386–25395 (2020).
11. PA Alpert, J Dou, P Corral Arroyo, al, Photolytic radical persistence due to anoxia in viscous aerosol particles. *Nat Commun* **12**, 1769 (2021).
12. D Piacentini, G Falasca, S Canepari, L Massimi, Potential of pm-selected components to induce oxidative stress and root system alteration in a plant model organism. *Environ. international* **132**, 105094 (2019).
13. L Massimi, et al., Spatial mapping and size distribution of oxidative potential of particulate matter released by spatially disaggregated sources. *Environ. Pollut.* **266**, 115271 (2020).
14. MA Frezzini, G Di Iulio, C Tiraboschi, S Canepari, L Massimi, A new method for the assessment of the oxidative potential of both water-soluble and insoluble pm. *Atmosphere* **13**, 349 (2022).
15. G Simonetti, et al., Oxidative potential of particulate matter components generated by specific emission sources. *J. Aerosol Sci.* **126**, 99–109 (2018).

16. C Molina, et al., Airborne aerosols and human health: Leapfrogging from mass concentration to oxidative potential. *Atmosphere* **11**, 917 (2020).
17. MA Frezzini, N De Francesco, L Massimi, S Canepari, Effects of operating conditions on pm oxidative potential assays. *Atmospheric Environ.* **268**, 118802 (2022).
